# FishCam: A low-cost open source autonomous camera for aquatic research

**DOI:** 10.1016/j.ohx.2020.e00110

**Published:** 2020-05-23

**Authors:** Xavier Mouy, Morgan Black, Kieran Cox, Jessica Qualley, Callum Mireault, Stan Dosso, Francis Juanes

**Affiliations:** a School of Earth and Ocean Sciences, University of Victoria, 3800 Finnerty Road, Victoria, BC V8P 5C2, Canada; b JASCO Applied Sciences, 2305-4464 Markham Street, Victoria, BC V8Z 7X8, Canada; c Biology Department University of Victoria, 3800 Finnerty Road, Victoria, BC V8P 5C2, Canada; d Geography Department, Memorial University of Newfoundland, P.O. Box 4200, St. John’s, NL A1C 5S7, Canada

**Keywords:** Biodiversity, Citizen science, Education, Fish monitoring, Raspberry Pi, Remote underwater video

## Abstract

We describe the “FishCam”, a low-cost (<500 USD) autonomous camera package to record videos and images underwater. The system is composed of easily accessible components and can be programmed to turn ON and OFF on customizable schedules. Its 8-megapixel camera module is capable of taking 3280 × 2464-pixel images and videos. An optional buzzer circuit inside the pressure housing allows synchronization of the video data from the FishCam with passive acoustic recorders. Ten FishCam deployments were performed along the east coast of Vancouver Island, British Columbia, Canada, from January to December 2019. Field tests demonstrate that the proposed system can record up to 212 h of video data over a period of at least 14 days. The FishCam data collected allowed us to identify fish species and observe species interactions and behaviors. The FishCam is an operational, easily-reproduced and inexpensive camera system that can help expand both the temporal and spatial coverage of underwater observations in ecological research. With its low cost and simple design, it has the potential to be integrated into educational and citizen science projects, and to facilitate learning the basics of electronics and programming.

## Graphical abstract

**Figure:**
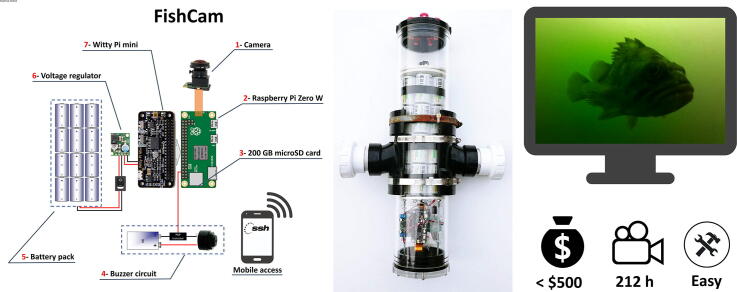

## 1 Hardware in context

**Specifications table:**

**Table:** 

**Hardware name**	FishCam
**Subject area**	• Biological Sciences
	• Environmental Sciences
	• Educational Tools and Open Source Alternatives to Existing Infrastructure
**Hardware type**	• Imaging tools
	• Field measurements and sensors
**Open source license**	Creative Commons Attribution-ShareAlike license
**Cost of hardware**	< $500.00 USD
**Source file repository**	• OSF: https://doi.org/10.17605/OSF.IO/8BGHA
	• GitHub: https://github.com/xaviermouy/FishCam.git

Underwater cameras are essential equipment for studying aquatic environments. They can be deployed in a variety of ways and in different habitats to monitor and observe marine or freshwater flora and fauna. Remote underwater video (RUV) cameras are autonomous cameras attached to small platforms that are typically deployed on the seabed for several hours. RUVs have been used successfully to study fish diversity, abundance and behavior, and, when equipped with a pair of cameras, can estimate fish sizes [1], [2], [3]. They have the advantage of observing the underwater environment without human disturbance but have limited temporal coverage. Camera systems can also be deployed permanently on the seabed, connected to linked networks such as Ocean Networks Canada’s NEPTUNE and VENUS cabled observatories [4]. These installations have limited spatial coverage but provide substantially longer time series since they receive power from and transmit data to shore stations via cable [5]. When mounted on mobile platforms, underwater cameras can cover larger spatial areas. Systems tethered on sleds towed on the seabed by surface vessels are used to map benthic habitats [6]. Cameras attached inside fish trawl nets count and measure fish for fisheries applications [7]. Remotely operated vehicles (ROVs) are also equipped with cameras and have been used to assess fish assemblages [8] and map hydrothermal vent fauna [9]. These cameras are expensive to purchase, operate and maintain, and consequently are accessible only to a limited number of research groups. However, the emergence of low-cost microcontrollers, single-board computers and sensors are creating new possibilities for data collection [10]. Scientists are increasingly able to design their own low-cost instruments tailored to their specific needs. For example, Favaro et al. [11] developed a camera system to study deep-water animals for under 3,000 USD. The system is able to record up to 13 h of video data at a time. Williams et al. [12] created an underwater stereo-camera trap using off-the-shelf commercial point-and-shoot cameras and a Raspberry Pi single board computer. Their system cost 1,800 USD and captured time-lapse images (not video) with a maximum autonomous deployment time of about 6 h. Wilby et al. [13] designed an acoustically-triggered underwater camera system for 5,500 USD. Their instrument was composed of six video cameras and a hydrophone, and had a battery run-time of 180 h, assuming actual video recording of 5% of the time (i.e. 9 h). Despite being more affordable, the autonomy of these systems is restricted to only a few hours and sometimes limited to images only. Finally, not all studies provide schematics and instructions required to build their systems, which limits the accessibility to other users.

We aimed to address these limitations by developing an underwater camera design that is low cost (<500 USD), has an autonomy of several days, and is relatively simple to construct via our publicly available schematics. The system was designed to help catalog fish sounds in the wild [14], but can find applications in a variety of research fields. Requirements for the camera system were to (1) be low cost (<500 USD), (2) be autonomous, (3) be able to record both video and still images for at least several days, (4) have the ability to be turned ON and OFF on duty cycles, and (5) be easily constructed using readily available components.

## 2 Hardware description

### 2.1 Electronic design

**Fig. 1:**
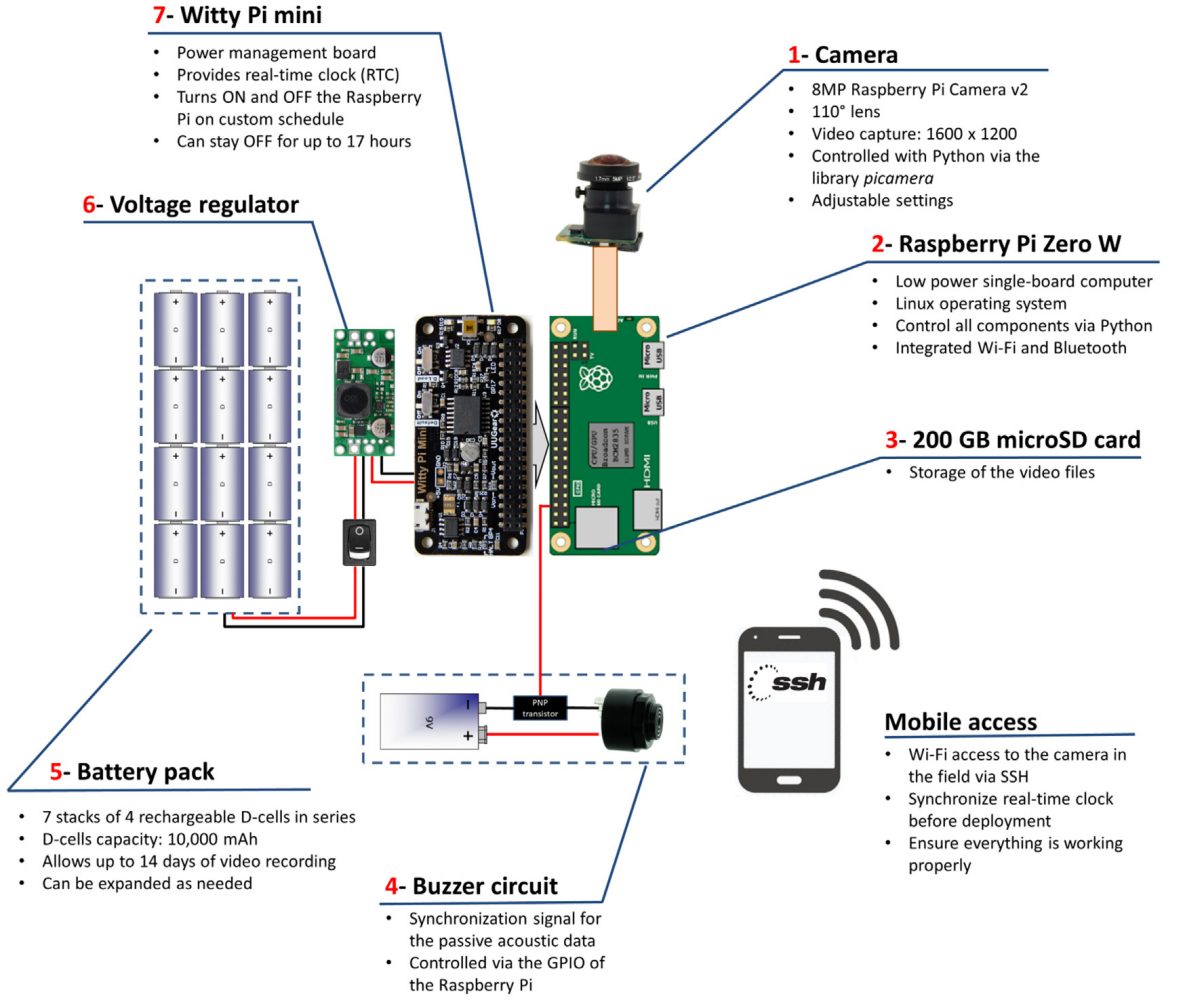
Overview of the FishCam electronic components and functionalities.

The core of the FishCam is made of a Raspberry Pi Zero W low-cost single-board computer (Raspberry Pi Foundation, Cambridge, United Kingdom), a WittyPi power management board (UUGear, Prague, Czech Republic) and low-cost electronics (Fig. 1). The Raspberry Pi Zero W has a small footprint (65 mm × 30 mm), uses a Linux operating system and can be configured as a desktop computer by connecting it via the HDMI and USB ports to an external monitor, keyboard and mouse. Its embedded WiFi card allows wireless access to the system, which provides a convenient way to monitor the FishCam in the field with a mobile device (e.g. ensure the camera is working properly just prior to deployment). All data and operating systems are stored on a 200 GB microSDXC card. Using cards with a UHS Speed Class of 1 (USH-1) provides sufficient transfer speed to record video data in the h264 format. An 8 MegaPixel Raspberry Pi camera module v2 fitted with a 110^°^ lens (Blue Robotics, Torrance, USA) is connected to the Raspberry Pi via its FPC connector and can capture high-definition video and digital still images. The WittyPi Mini board, connected via the Raspberry Pi general-purpose input/output (GPIO) pins, provides a Real Time Clock and allows the FishCam to be turned ON and OFF in custom duty cycles. The system is powered by a battery pack comprised of seven stacks of four EBL D-Cell rechargeable batteries in series, providing a total capacity of 70,000 mAh. A Pololu (Las Vegas, USA) 5 V 2A Step-Up/Down regulator is placed between the output of the battery pack and the input of the WittyPi Mini to increase or decrease the voltage as necessary to produce fixed 5 V power. This makes the FishCam more stable, extends its autonomy, and allows the use of either rechargeable or non-rechargeable batteries. Finally, an external buzzer circuit connected via the GPIO pins can produce an acoustic signal on-demand to time synchronize the video data from the FishCam with data from other audio recording instruments deployed in the vicinity.

### 2.2 Mechanical design

**Fig. 2:**
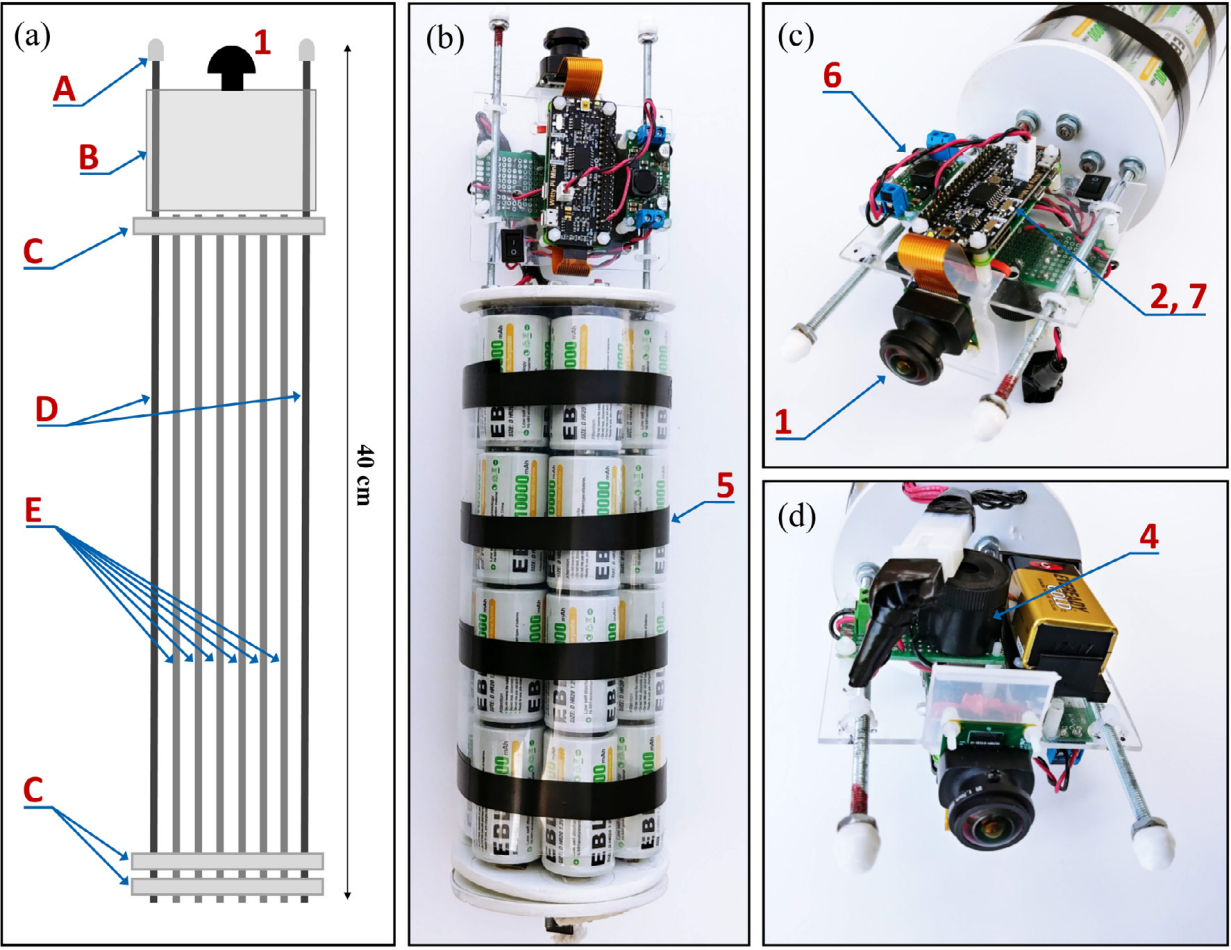
Internal frame supporting the FishCam electronic components: (a) Diagram and (b) photograph of the internal camera components, (c) photograph of the main core electronic circuit, and (d) buzzer circuit. Numbers indicate the electronic components defined in Fig. 1. Letters identify the main mechanical components described in Section 2.2.

The frame holding all the electronics of the FishCam is made out of consumer-grade items (identified by letters in Fig. 2) that are typically found at local hardware stores or from popular online retailers. The main components are eight 8–32 aluminum threaded rods (D, E) bolted to three 4” separator disks (C) and a 3” × 3.75” × 0.08” clear acrylic sheet (B). The six short rods (E) serve as holders for the D-cell batteries (5), while the two longer rods (D) extend up to the tip of the camera lens (1) to hold the acrylic sheet (B) and support the weight of the FishCam when placed upside down without damaging the camera sensor. Rubber padding (A) is added at the end of each of these rods to avoid scratching the front-view window of the FishCam pressure housing. The acrylic sheet (B) holds all the electronics using silicon M3 mounts and is secured to the rods (D) with small zip ties. The separator disks (C), cut from virgin-grade PVC vinyl ceiling tiles, have spring connectors and wires glued on one side to connect each D-cell stack in parallel. The 4” diameter of the disks fit seven D-cell stacks and maintain stability of the internal frame inside the pressure housing. A laminated plastic sheet wrapped around the battery pack and secured with electrical tape holds the batteries in place. The Raspberry Pi, WittyPi and voltage regulator are placed on one side of the acrylic sheet (Fig. 2c), while the PCB board with the buzzer circuit is placed on the opposite side (Fig. 2d). The sheet is perforated at several places to allow wires to connect components from both sides.

**Fig. 3:**
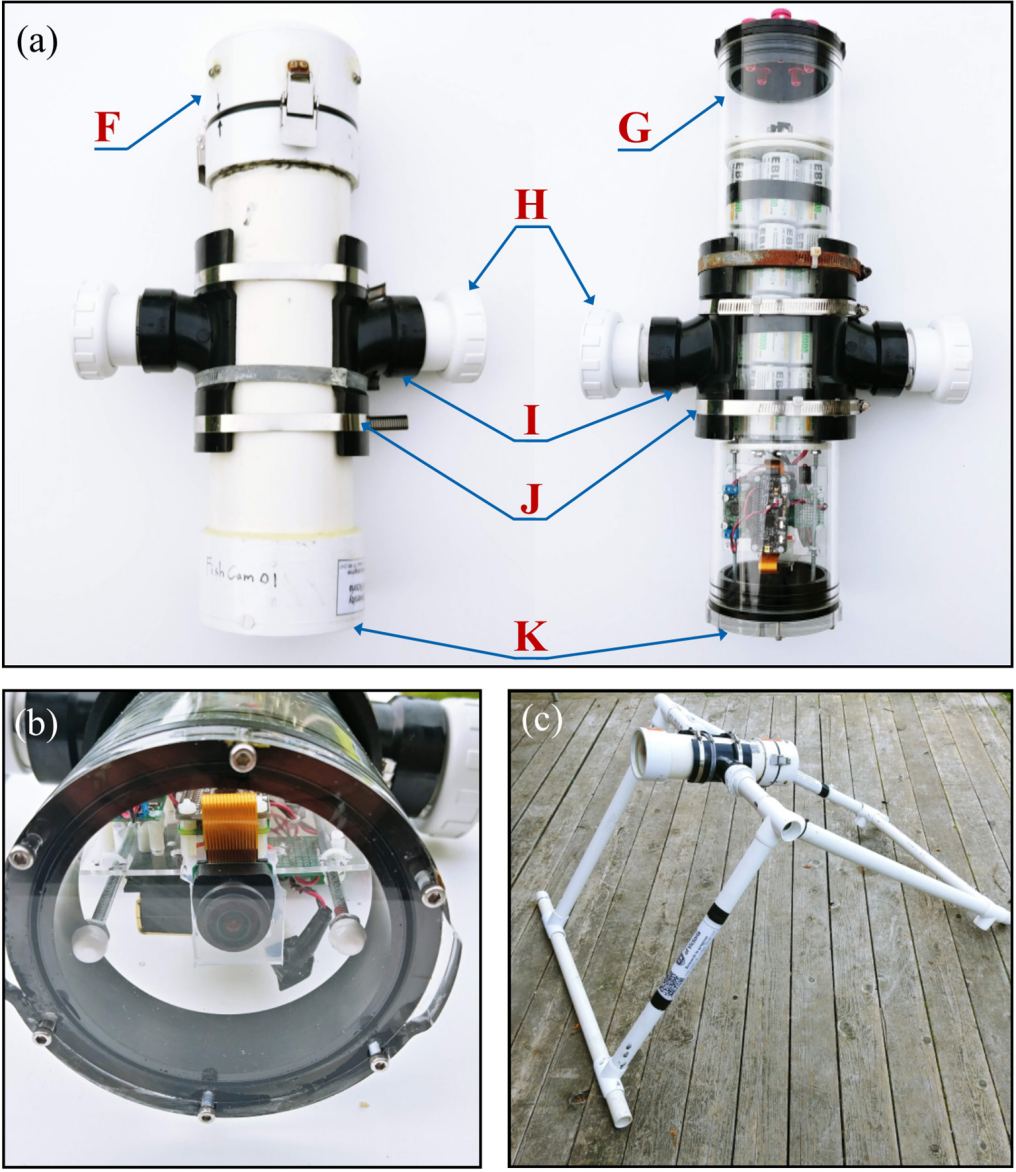
External components of the FishCam: (a) homemade (left) and commercially made (right) pressure housings with PVC attachments, (b) front plexiglass window, (c) example of PVC frame for holding the FishCam. Letters define the mechanical components described in Section 2.2.

The internal frame with all the electronics is inserted in a pressure housing in order to be deployed in an aquatic environment. Two possible pressure housing designs (one homemade and the other commercially available) were tested and their key components are identified by letters in Fig. 3. The first pressure housing (F) is made of Schedule 40 PVC pipe and based on [15] but with a longer tube. This housing is inexpensive (~100 USD), made of readily available parts, and was pressure tested to water depths of at least 30 m (50 psi). The second pressure housing (G) is assembled from components available from Blue Robotics. It is rated to 100 m depth, costs ~260 USD, and has five watertight openings on the end-cap to accommodate external connections. Both housings have a front-view window on one end (K, Fig. 3b) for the camera and an internal diameter of 4”, which allows a snug fit with the internal FishCam frame.

Both pressure housings are fitted with two 4” × 4” × 2” ABS Tee fittings cut in half along the 4” section (I) and held together with three stainless steel collars (J). A 1–1/2” PVC union fitting (H) is cemented to the 2” end of the tee fittings via a small section of 1–1/2” PVC pipe and a 2” to 1–1/2” bushing. This allows the FishCam to be easily attached/detached vertically or horizontally on any structure made out of 1–1/2” PVC pipe. The angle of the FishCam can easily be adjusted by loosening and tightening the screw of the PVC union fittings. Fig. 3c shows an example of a simple PVC frame design to deploy the FishCam on the seabed.

### 2.3 Software

The FishCam is programmed via the Linux operating system (OS) of the Raspberry Pi. We used the Linux distribution Raspbian Buster with desktop [16], which has minimal software already installed, leaving more storage space for video data on the microSD card. In the initial setup of the OS, only the necessary software is installed manually. Once deployed, the FishCam runs the OS headless (i.e. without the graphical interface) to minimize computational overhead.

The duty cycle of the FishCam is controlled via the WittyPi. Upon initial installation of the WittyPi board on the Raspberry Pi, the scheduling software is installed following the user manual from the manufacturer [17]. The duty cycle is defined in a text file describing the start time and duration of each ON and OFF sequence. In all our field tests, because no external lights were used, the FishCam was put in sleep mode during night time to save battery life. To avoid having data gaps due to potential OS malfunctions (freezing), the FishCam was set to reboot every 4 h. Duty cycle sequences can be customized to fit multiple research purposes. Before deployment, the Real Time Clock of the WittyPi must be synchronized with the Raspberry Pi clock using the manufacturer’s interface. The WittyPi mini has no external battery but powers the Real Time Clock using a supercapacitor which can only remember the time for 17 h without external power. As a consequence, the duty cycle programmed on the Witty Pi mini should not have an OFF time greater than 17 h.

Operational mode settings and acquisition of data from the camera sensor are controlled in Python using the well-documented picamera library [18]. Many camera settings can be adjusted, including, but not limited to, resolution, frame rate, exposure and ISO. In all our field tests, the camera was set to record video on 5-min h264 files at 10 frames per second (maximum rate available: 30 frames per second) with a resolution of 1600 × 1200 pixels at ISO 400. While the mjpeg video format is also available, here we chose the h264 format as it generates smaller files and requires less GPU resources. The python script runs automatically once the OS starts using a job scheduler (i.e. Crontab). The buzzer circuit is also activated via Python using the library RPi.GPIO [19] and emits a short sequence of beeps when the OS starts and video data acquisition has successfully begun. In the field, not hearing the buzzer sequence after turning ON the FishCam indicates an issue with the data acquisition. Buzzer sequences are customizable, so that instruments can be differentiated acoustically if several FishCams are deployed at the same location. The buzzer circuit can be turned off if not required.

Wireless access to the FishCam is possible by activating the WiFi card of the Raspberry Pi and enabling SSH connections to the OS. Freely available phone applications such as RaspController [20] can be used to configure or monitor the FishCam in the field (e.g. synchronize the Real Time Clock, preview or live stream of video data being acquired, monitor CPU usage, etc.).

## 3 Design files

### 3.1 Design files summary

**Table:** 

Design filename	File type	Open source license	Location of the file
FishCam overview	Figure (jpg)	CC BY-SA	Available with the article (Fig. 1)
Buzzer circuit	Figure (jpg)	CC BY-SA	Available with the article (Fig. 12)
Components placement	Figure (jpg)	CC BY-SA	Available with the article (Fig. 15)
FishCam_scripts	zip	CC BY-SA	Source file repository and Github

• FishCam overview: A diagram showing the different electronic components of the FishCam.
• Buzzer circuit: Schematic of the buzzer electronic circuit.
• Components placement: Diagram showing how each component is placed on the mounting plate.
• FishCam_scripts: Zip file containing all the code necessary to operate the FishCam.

## 4 Bill of materials

**Table:** 

Designator	Component	Number	Cost per unit currency	Total cost	Source of materials	Material type
P1	D-Cell rechargeable batteries. 10,000 mAh. 1.2 V Ni-Mh	28	$5.33 USD	$149.30 USD	Amazon	Other
P2	ON/OFF rocker switch	1	$1.48 USD	$1.48 USD	BC-Robotics	Other
P3	Raspberry Pi zero W	1	$14.95 USD	$14.95 USD	Ameridroid	Other
P4	2 × 20 pin breakaway male header	1	$1.48 USD	$1.48 USD	BC-Robotics	Other
P5	Witty Pi Mini	1	$16.48 USD	$16.48 USD	UUGEAR	Other
P6	Camera adapter cable for Raspberry Pi Zero	1	$5.95 USD	$5.95 USD	Ameridroid	Other
P7	Raspberry Pi camera module v2 with wide angle lens	1	$49.00 USD	$49.00 USD	Blue Robotics	Other
P8	5 V 2A Step-Up/Down regulator	1	$13.54 USD	$13.54 USD	BC-Robotics	Other
P9	200 GB MicroSDXC UHS-I memory card	1	$26.99 USD	$26.99 USD	Amazon	Other
P10	M2.5 male + female hex nylon spacer, standoff, bolt, screw, nuts	1	$10.55 USD	$10.55 USD	Amazon	Other
P11	Micro JST 1.25MM 2-Pin male + female connectors	1	$1.00 USD	$1.00 USD	BC-Robotics	Other
P12	Assortment of electronics wires	1	$1.00 USD	$1.00 USD	Amazon	Other
P13	Set of male + female 2-pin Molex connectors	1	$1.60 USD	$1.60 USD	Amazon	Other
P14	Plastic egde protector	1	$2.00 USD	$2.00 USD	Amazon	Other
P15	PCB board prototype	1	$0.60 USD	$0.60 USD	Amazon	Other
P16	PNP transistor 2N3904	1	$0.25 USD	$0.25 USD	Amazon	Other
P17	10 kOhm resistor	1	$0.06 USD	$0.06 USD	Amazon	Other
P18	9 V battery	1	$3.49 USD	$3.49 USD	Amazon	Other
P19	9 V battery holder	1	$0.40 USD	$0.40 USD	Amazon	Other
P20	Large Piezo Alarm - 3KHz	1	$3.25 USD	$3.25 USD	RobotShop	Other
P21	D cell battery spring and plate connectors	14	$0.32 USD	$4.48 USD	Digikey	Other
P22	OPTIX 0.08” × 8” × 10” clear acrylic sheet	1	$3.00 USD	$3.00 USD	Lowe’s	Other
P23	3’ 8–32 threaded rods	4	$1.5 USD	$6.00 USD	Lowe’s	Other
P24	#8 washers	20 (1 box)	$3.23 USD	$3.23 USD	Lowe’s	Other
P25	8–32 nuts	20 (1 box)	$2.25 USD	$2.25 USD	Lowe’s	Other
P26	#8 lock washers (1 box)	20	$2.99 USD	$2.99 USD	Lowe’s	Other
P27	#8 cap nuts (1 box)	2	$2.99 USD	$2.99 USD	Lowe’s	Other
P28	Screw protector	2	$0.50 USD	$1.00 USD	Lowe’s	Other
P29	5-mil 11” × 17” laminated plastic sheet	1	$1.89 USD	$1.89 USD	Staples	Other
P30	2’ × 4’ virgin-grade PVC vinyl ceiling tile	1	$12.06 USD	$12.06 USD	Lowe’s	Other
P31	Roll of 22-gauge metal hanger strap	1	$4.89 USD	$4.89 USD	Lowe’s	Other
P32	4” × 0.1” miniature cable ties	4	$4.52 USD	$4.52 USD	Lowe’s	Other
P33	Pressure housing option 1: PVC housing from [15]	1	$100.00 USD	$100.00 USD	Any plumbing store	Other
P34	Pressure housing option 2: Watertight enclosure for ROV/AUV (4” Series)	1	$233.00 USD	$233.00 USD	Blue Robotics	Other
P35	4” × 4” x-2” tee fittings (ABS)	2	$9.52 USD	$19.04 USD	Home Depot	Other
P36	2” × 1–1/2” flush bushing fittings (ABS)	2	$1.48 USD	$2.96 USD	Lowe’s	Other
P37	Slip PVC union fitting	2	$5.29 USD	$10.58 USD	Pool and Hot Tub Depot	Other
P38	7” piece of 1–1/2” PVC tube schedule 40	2	$3.56 USD	$3.56 USD	Lowe’s	Other
P39	6” stainless steel adjustable clamps	3	$1.83 USD	$5.49 USD	Lowe’s	Other

## 5 Build instructions

### 5.1 Assembling the electronics

#### 5.1.1 Main camera circuit

1. Solder the breakaway male header (P4) to the Raspberry Pi (P3, Fig. 4).

**Fig. 4:**
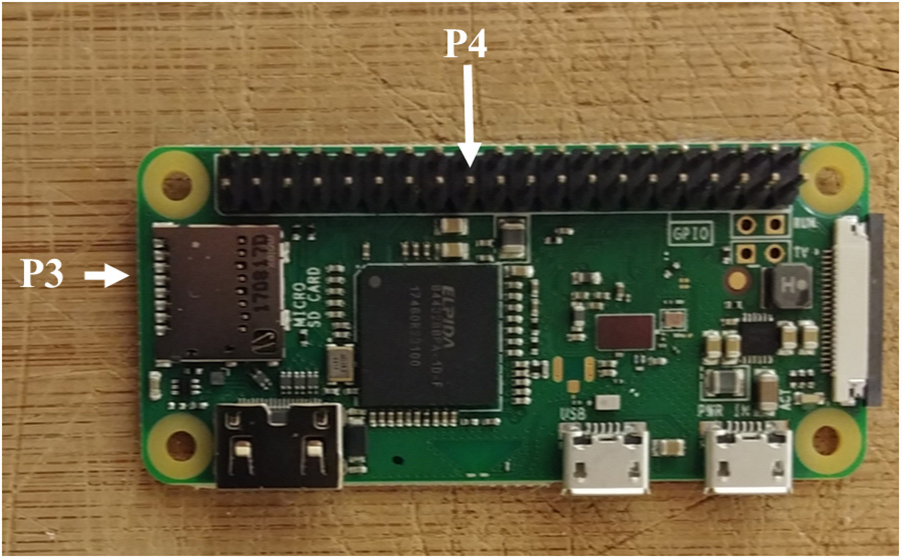
Raspberry Pi zero with male header.
2. Solder the micro JST male connector (P11) to the battery port of the Witty Pi (P5), circled in red bellow (Fig. 5).

**Fig. 5:**
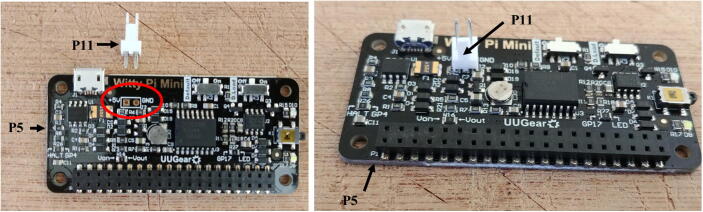
Soldering of the JST male connector on the Witty Pi.
3. There are two switches on the Witty Pi board. Ensure that the switch *Default* is ON and *D-Load* is OFF.
4. Take four silicon mounts (P10) and insert them through each mounting hole of the Raspberry Pi (Fig. 6).

**Fig. 6:**
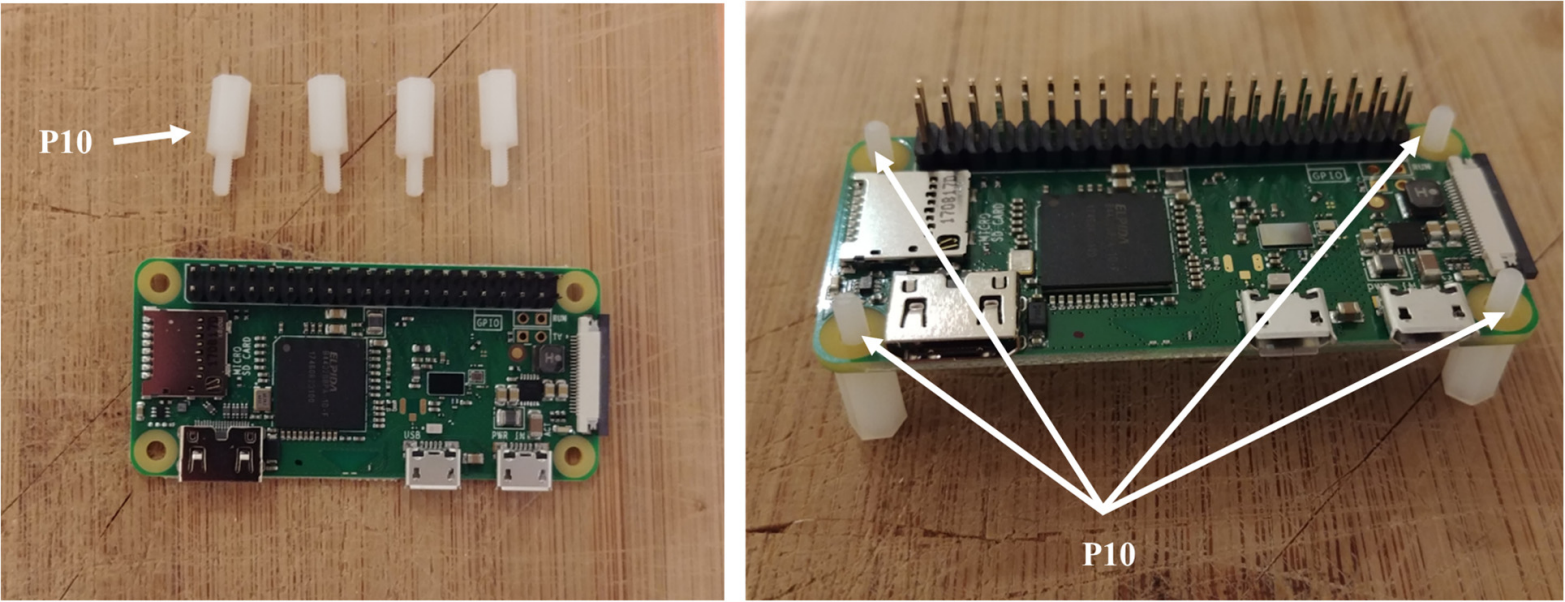
Installation of the silicon mounts.
5. Take four more silicon mounts (P10) and screw them on top of the ones installed in step 4. Stack the Witty Pi on top of the Raspberry Pi via the male header (P4), and screw four silicon nuts on top of each mount to secure the two boards together (Fig. 7).

**Fig. 7:**
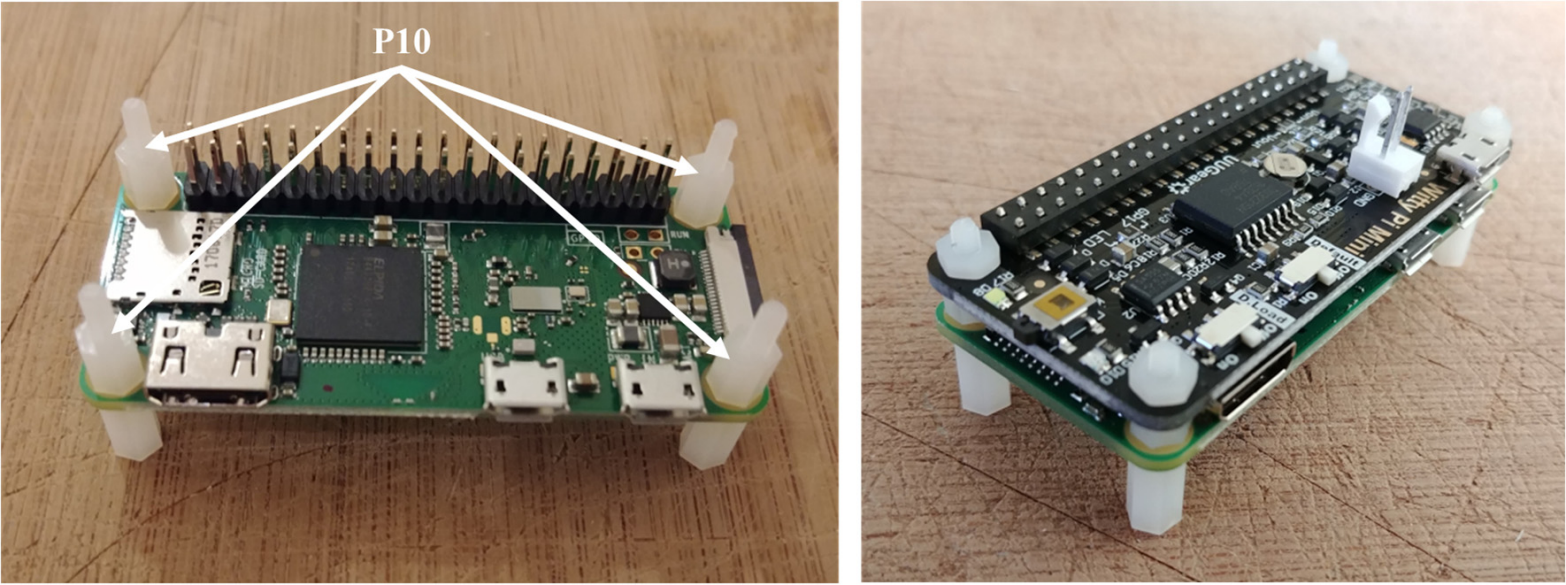
Installation of Witty Pi.
6. Solder the two terminal blocks (included with the regulator) to the input and output ports of the voltage regulator (P8, Fig. 8).

**Fig. 8:**
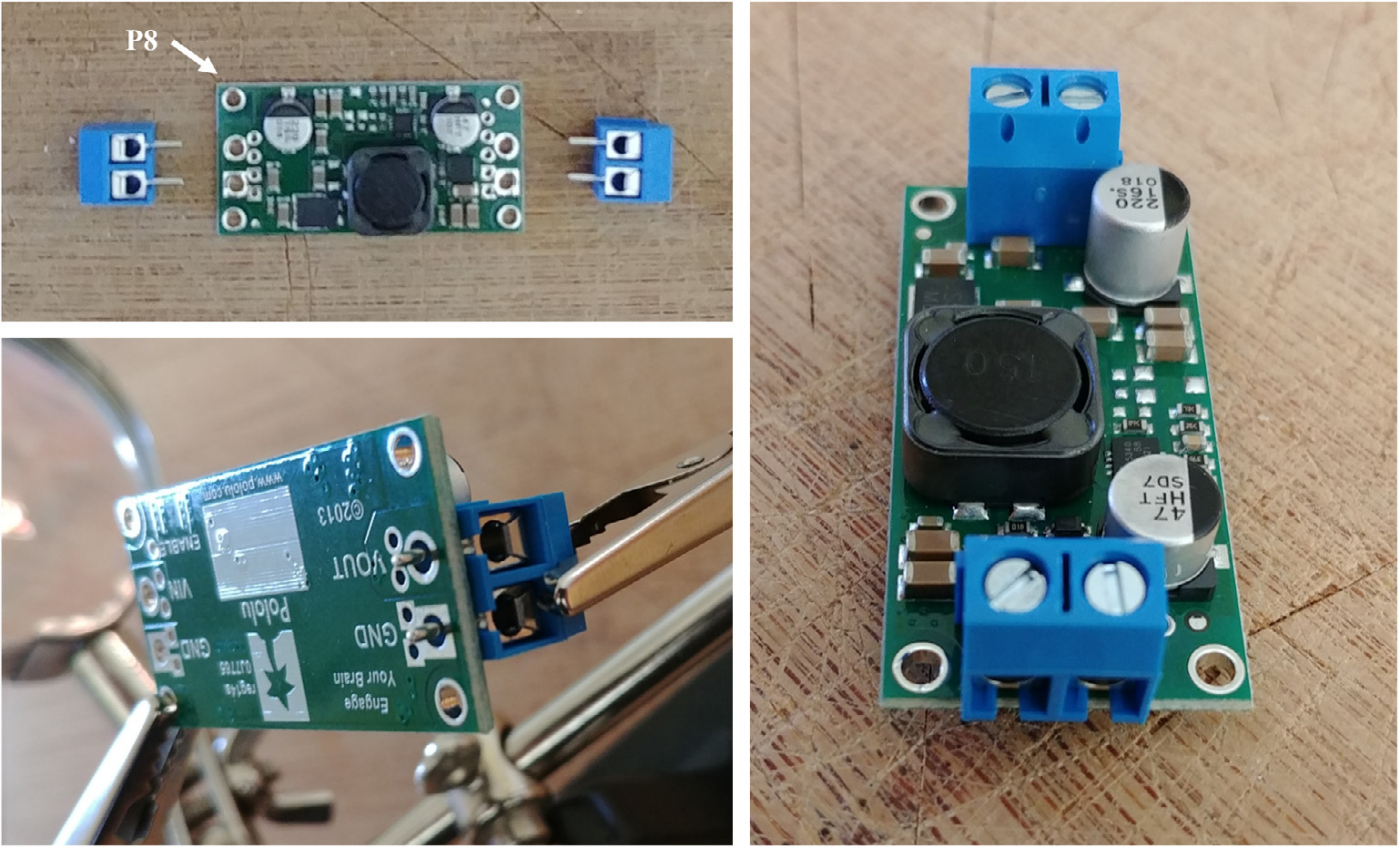
Preparation of the voltage regulator.
7. Cut and strip both ends of a red and a black wire (P12) about 10 cm long each. Solder one end of each wire to the micro JST female connector (P11). Ensure the red and black wires are connected to the JST connector in such a way that once the female connector is connected to the Witty Pi (via the male connector soldered in step 2), the red and black wires correspond to positive and negative ports of the Witty Pi. Connect the other end of the cables to the output of the voltage regulator via the terminal block (Fig. 9). Ensure the red and black wires are connected to the positive and negative outputs of the voltage regulator, respectively. Attach four silicon mounts (P10) to the mounting holes of the voltage regulator (Fig. 10).

**Fig. 9:**
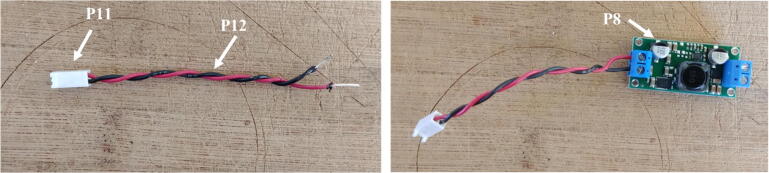
Installation of the JST female connector.

**Fig. 10:**
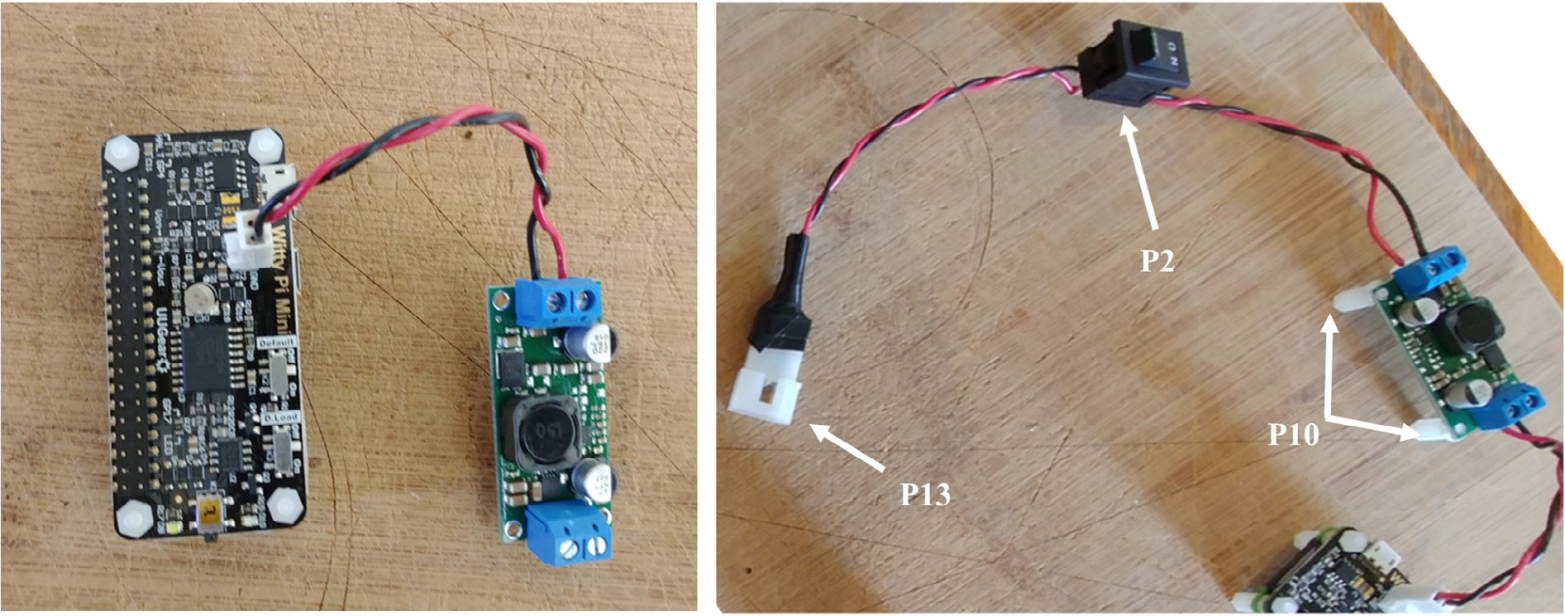
Assembly of Molex Connector, ON/OFF switch, voltage regulator, and Raspberry Pi.
8. Connect the voltage regulator (P8) to the Witty Pi (P5), using the micro JST connectors. Solder the Molex connector (P13), and the ON/OFF rocker switch (P2) to two wires going to the input block connector of the voltage regulator (Fig. 10).
9. Cut a small piece of plastic edge protector (P14) and drill two holes in it to attach the camera sensor (P7). Attach the camera sensor to the plastic edge protector with nylon screws and nuts (P10). Connect the ribbon cable (P6) to the camera sensor (P7, Fig. 11).

**Fig. 11:**
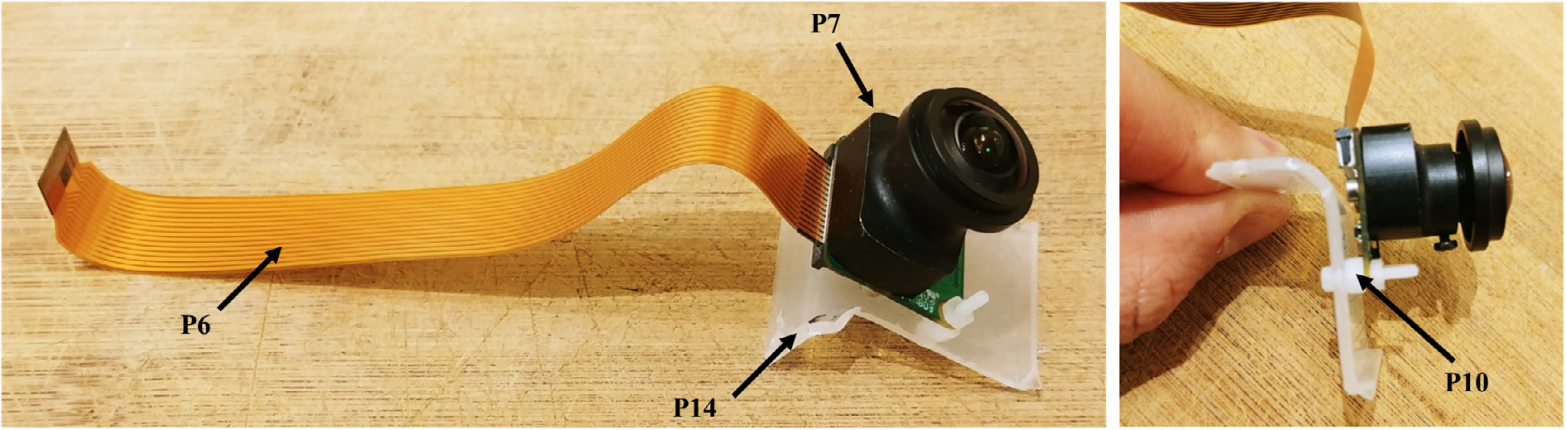
Installation of the camera sensor on its mount.

#### 5.1.2 Buzzer circuit

**Fig. 12:**
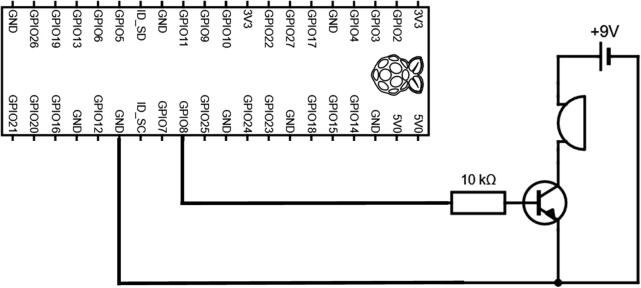
Electronic diagram of the buzzer circuit.

The buzzer circuit is composed of a piezo alarm, a resistor, a transistor, and a 9 V battery (Fig. 12). The circuit is connected to the pins GPIO-8 and Ground of the Raspberry Pi. The transistor acts as a switch that connects/disconnects the 9 V battery to the piezzo alarm. When the GPIO-8 logic pin is turned ON via a Python script, a voltage of 3.3 V from the Rapsberry Pi is delivered to the base of the transistor which closes the circuit and consequently turns ON the piezzo alarm. The Python script controlling the buzzer circuit is described in Section 6.6. The steps to assemble the buzzer circuit are:

1. Place and solder the electronic components P16, P17, and P20 on a 3.2 cm × 4.5 cm PCB prototyping board (P15) as shown in Fig. 13.

**Fig. 13:**
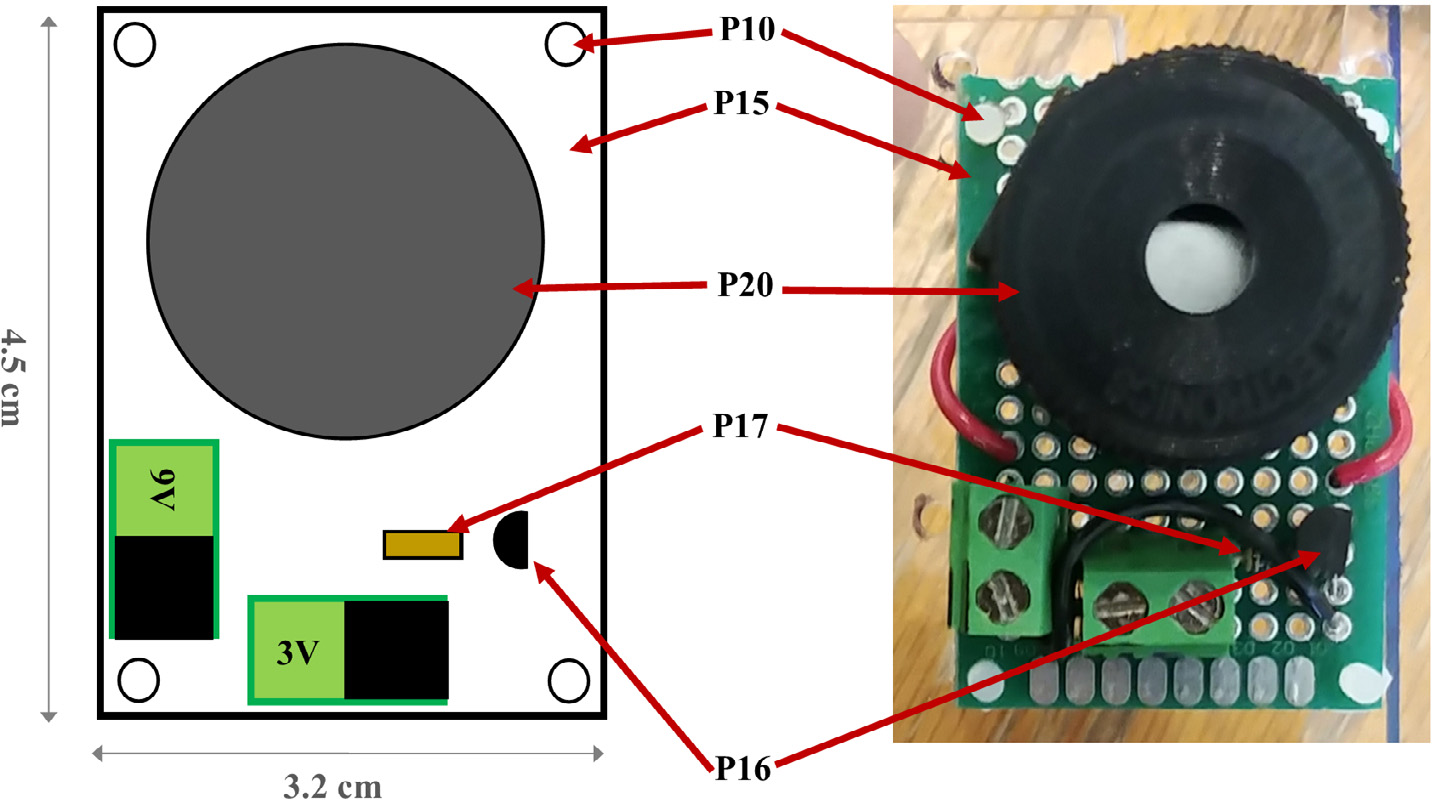
Buzzer circuit.
2. Solder electronics wires (P12) to connect the electronic components as shown in the electronic diagram in Fig. 12.
3. Attach four silicon mounts (P10) to each corner of the PCB board (Fig. 13)
4. Solder two wires to the battery holder (P19) and connect them to the “9 V” terminal block of the buzzer circuit (Fig. 14).

**Fig. 14:**
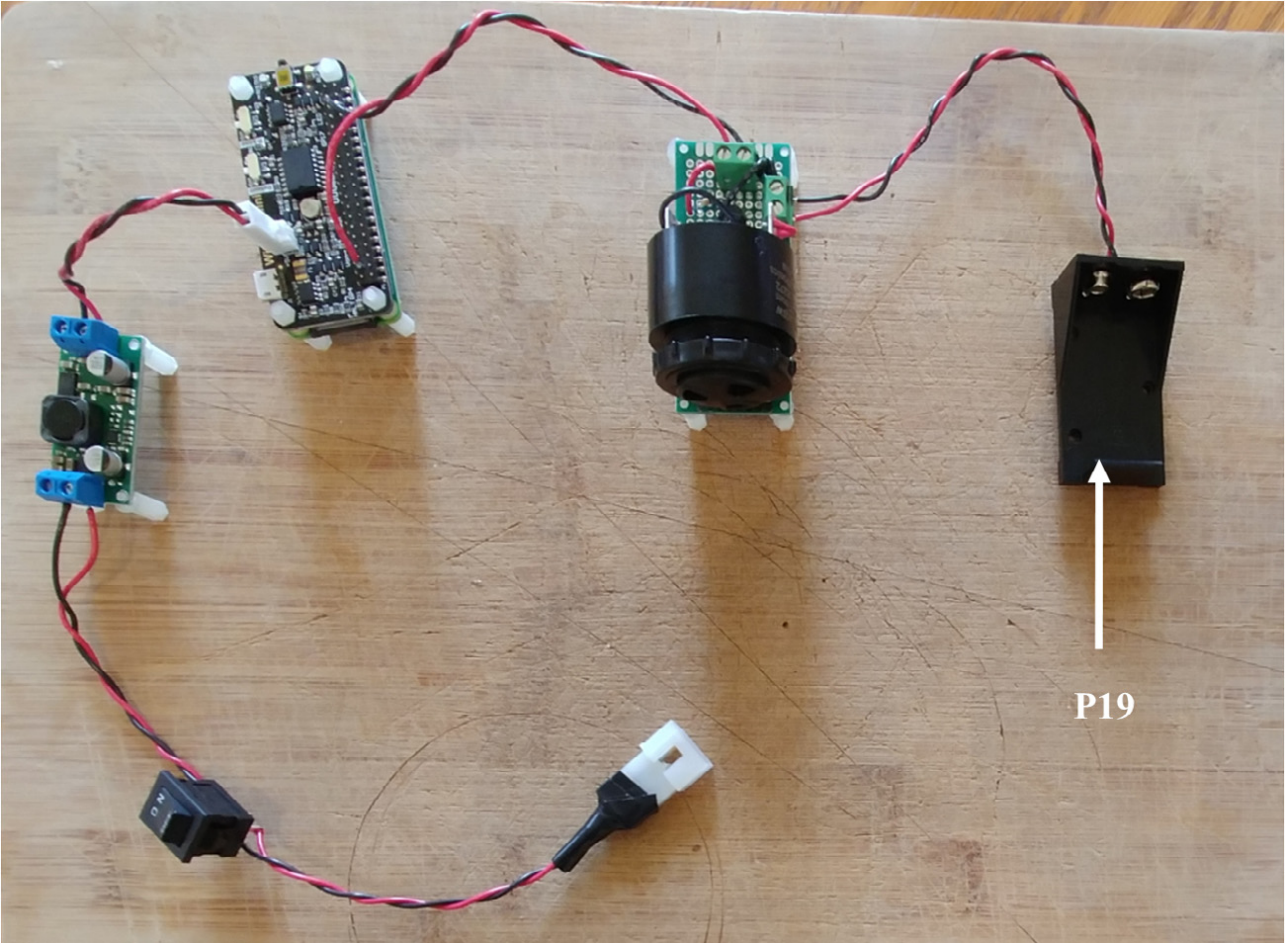
Assembly of all the electronic components.
5. Connect two wires between the “3 V” terminal block of the buzzer circuit and the GPIO pins “GND” and “GPIO-8” of the Raspberry Pi. The male header pins (P4) on the Raspberry Pi should be long enough to have the wires directly soldered on them (Fig. 14)
6. The buzzer circuit is now connected to the Raspberry Pi and can be controlled via a Python script (see Section 6.6).

#### 5.1.3 Assembly of the electronics on the mounting plate

**Fig. 15:**
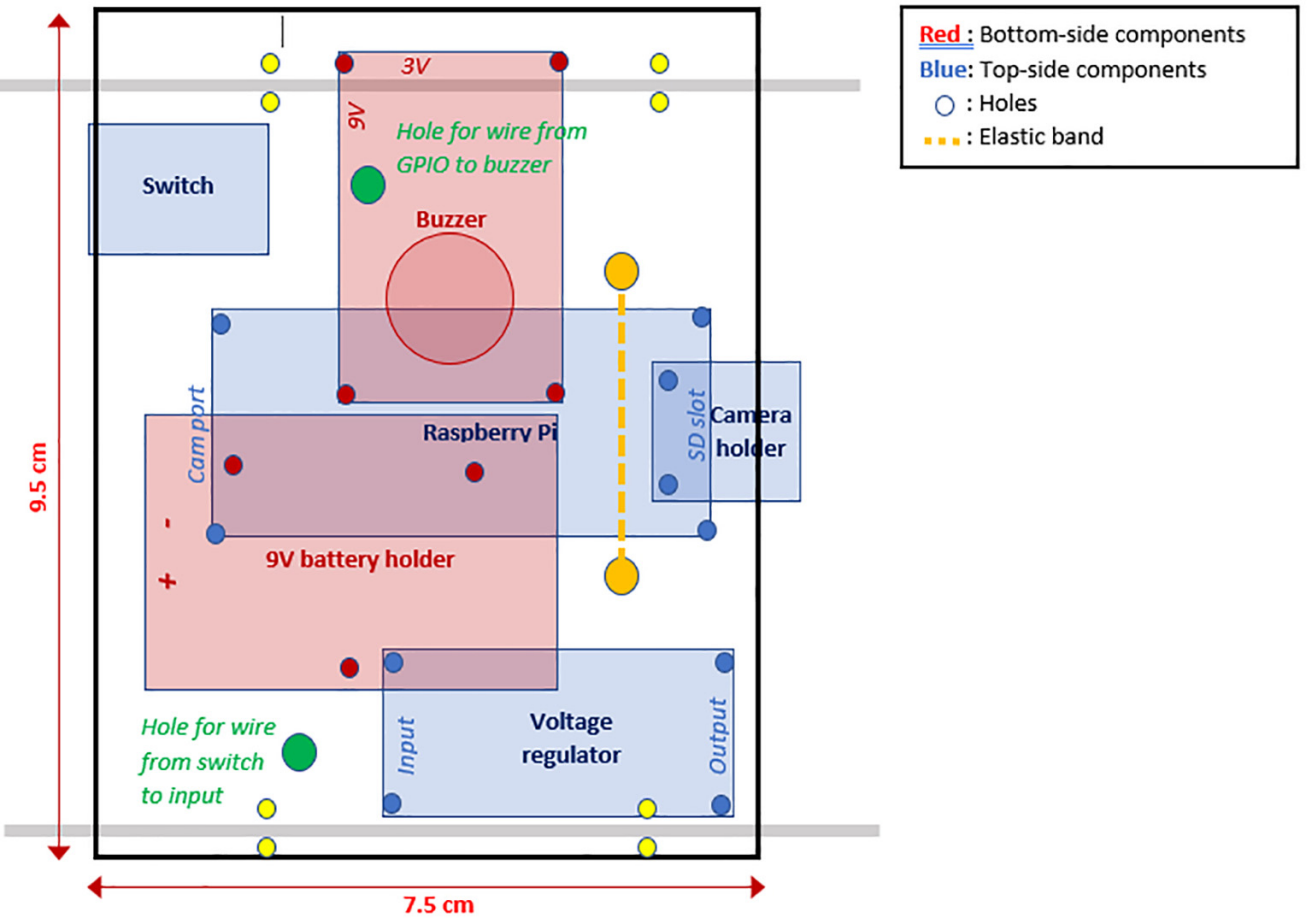
Placement of the electronic components on the mounting plate.

All the electronic components of the FishCam are attached to the mounting plate (P22) held in place by the aluminum rods of the internal frame. The diagram in Fig. 15 indicates the placement of each component. The blue symbols indicate components that are on the top-side of the mounting plate. Red symbols indicate components placed on the bottom-side of the mounting plate. Circles indicate mounting holes. The two bigger green circles indicate the holes for wires that connect the buzzer circuit to the GPIO pins of the Rapsberry Pi and the ON/OFF switch to the input of the voltage regulator. The two holes denoted by the orange circles allow the attachment of an elastic band (dashed orange line) that holds down the ribbon cable of the camera sensor. The four pairs of yellow circles indicate holes that are used to attach the mounting plate to the aluminum rods of the internal frame (horizontal grey lines) using small cable ties (P32).

**Fig. 16:**
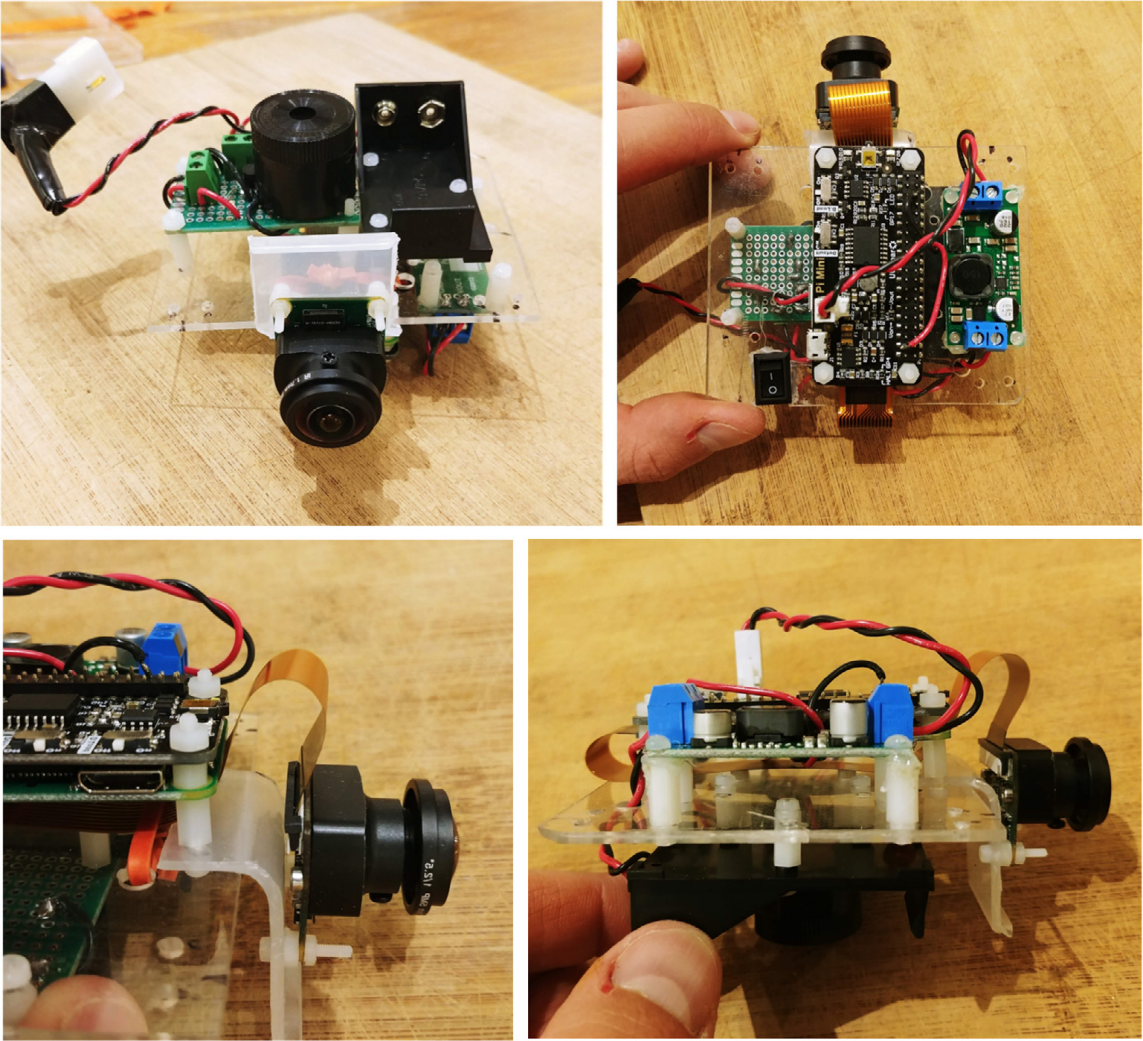
Electronic components installed on the mounting plate.

1. With a utility knife, cut a mounting plate of dimensions 7.5 cm × 9.5 cm out of the clear acrylic sheet (P22).
2. With a marker pen, mark on the mounting plate the locations of the ON/OFF switch and all of the holes.
3. Drill all the holes on the mounting plate with a drill and cut the rectangle of the switch with utility knife.
4. Tie a short elastic band through the holes indicated by the orange/yellow circles. (Fig. 15).
5. Temporarily disconnect all wires of the electronic components from the terminal blocks.
6. Attach the buzzer circuit and the 9 V battery holder on the bottom side of the mounting plate with the silicon screws (P10).
7. Attach the voltage regulator, the camera sensor (via the camera holder) and the Raspberry Pi on the top side of the mounting plate using the silicon screws (P10). The camera sensor should be placed such that the ribbon cable connector is on the top side of the mounting plate.
8. Connect the free end of the ribbon cable of the camera to the camera port of the Raspberry Pi. The cable must go underneath the Raspberry Pi and be held down by the elastic band.
9. Glue the ON/OFF switch to the mounting plate.
10. Reconnect all the wires to their respective terminal blocks (Fig. 16).

### 5.2 Building the internal frame

The internal frame holds the battery pack and the electronic components in the 4” diameter tube of the pressure housing. The steps to assemble the internal frame are as follows.

1. Draw a disc of diameter 4” on the PVC vinyl tile (P30) and cut it out with a utility knife (Fig. 17).

**Fig. 17:**
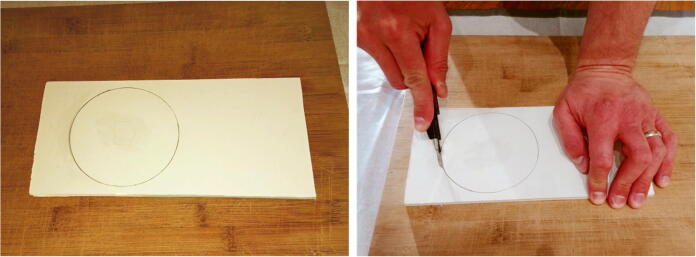
Cutting of the discs from the PVC vinyl tile.
2. Use sand paper to smooth the edges of the disc (Fig. 18). Ensure it fits perfectly into the tube of the pressure housing (i.e. a 4” diameter tube).

**Fig. 18:**
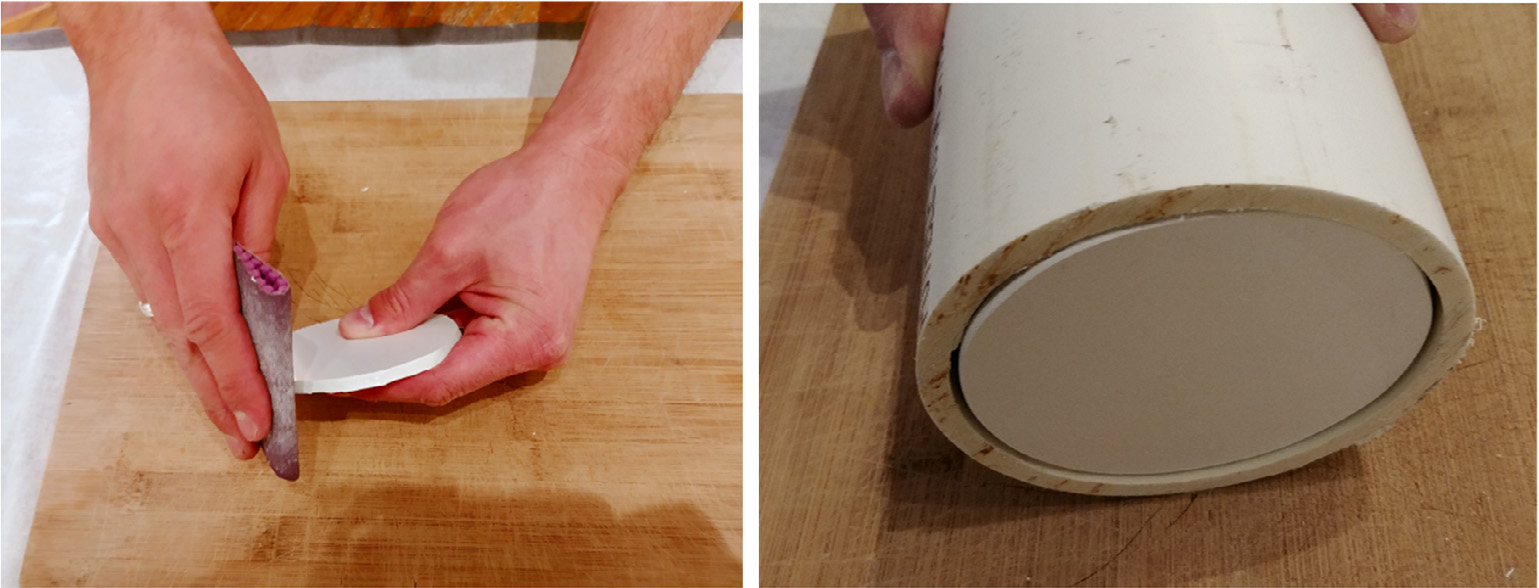
Sanding edges of the discs.
3. Place seven D-cell batteries perfectly centered on top of the disk. Place eight nails (or screws) between the D-cell batteries (Fig. 19).

**Fig. 19:**
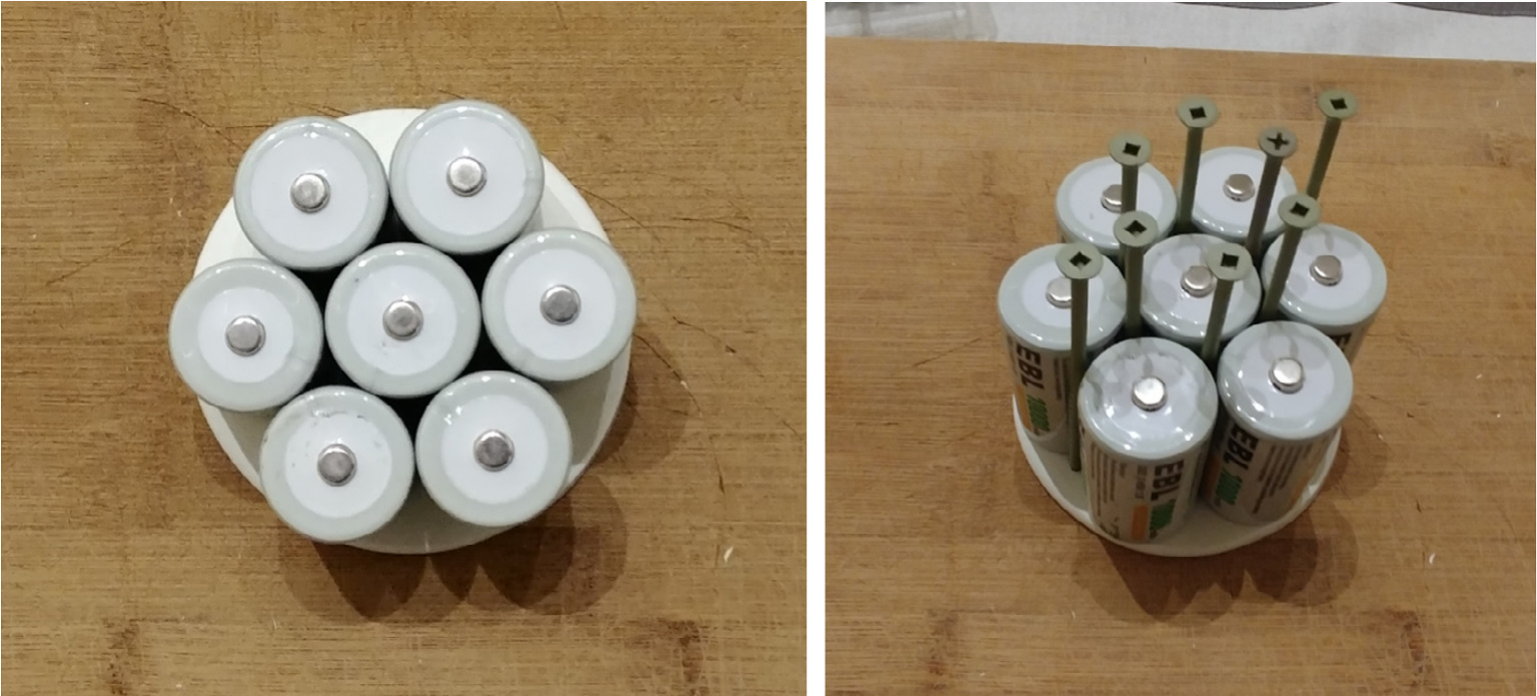
Locating holes to drill on the discs.
4. Gently tap on the nails with a hammer to leave a mark on the disk. Remove the batteries and nails, and drill holes at each mark on the disk. These holes will be used for holding the rods. The two holes indicated with the green circles will be for the longer rods extending up to the front-view window of the pressure housing. Drill two additional holes (indicated in red in Fig. 20 and referred to as “wire holes” in the following steps) large enough to fit seven wires (Fig. 20).

**Fig. 20:**
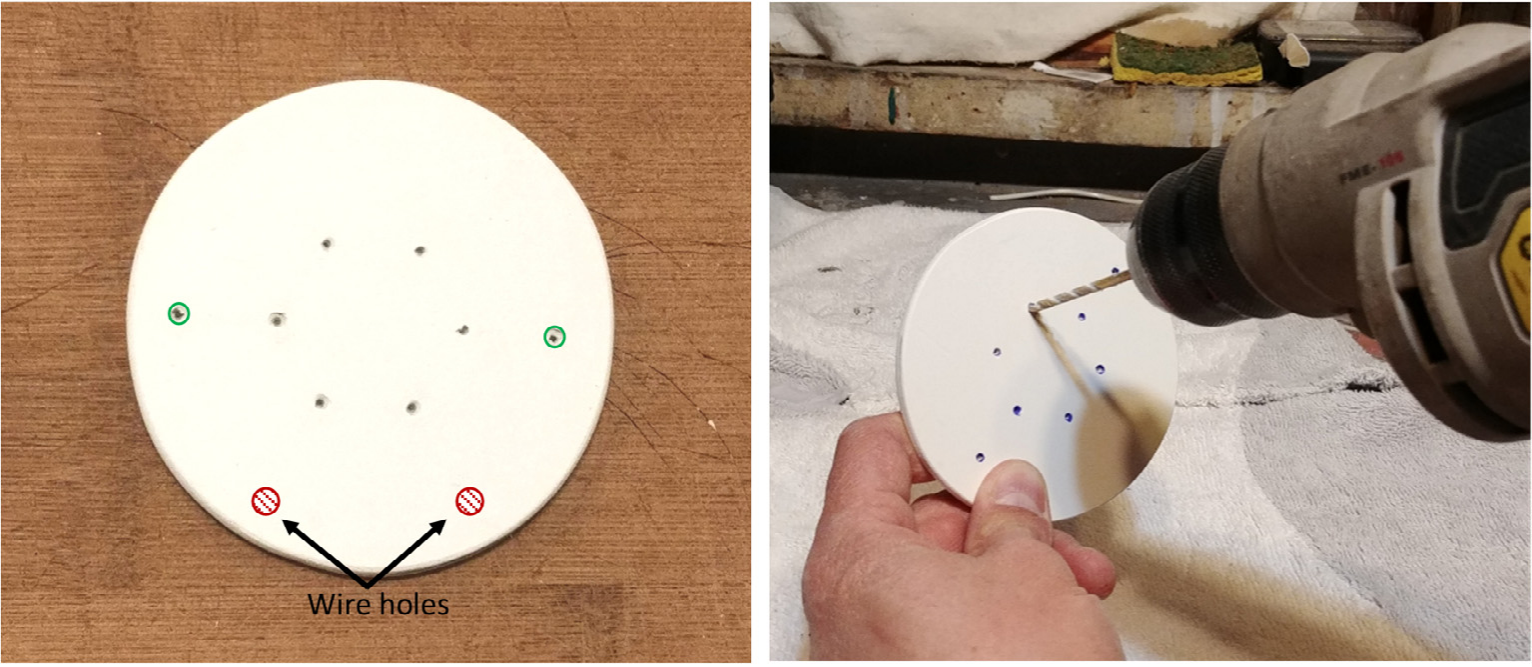
Drilling of the holes on the discs.
5. Repeat steps 1–4 twice to obtain three disks. The first disk, referred to as “positive disk”, will hold all the positive (+) battery connectors (P21 plates). The second disk, referred to as “negative disk”, will hold all the negative (−) battery connectors (P21 springs). The third disk, referred to as “connector-free disk”, will serve as a structural component and will not have any connectors attached to it. The connector-free disk should not have any wire holes drilled into it (i.e. red-labeled holes in Fig. 20).
6. Solder red wires to the seven positive battery connectors (P21 plates), glue them to the positive disk, and pass the free end of the wires through one of the wire holes (Fig. 21). The red wires should be ~ 40 cm long.

**Fig. 21:**
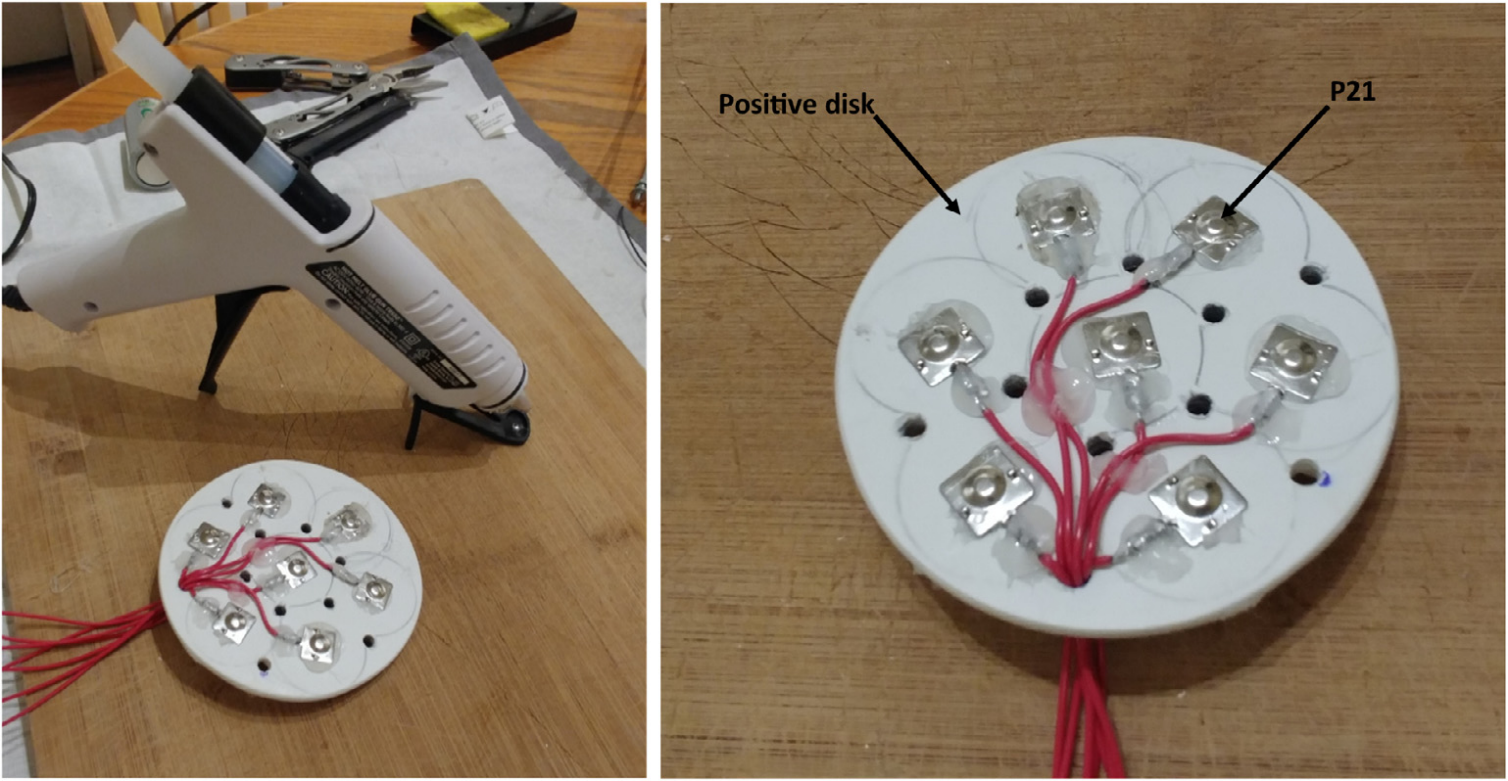
Battery connectors glued to the discs.
7. Repeat step 6, but this time using black wires and the negative battery connectors (P21 springs) on the negative disk. The black wires should be ~ 15 cm long.
8. With a metal saw cut six 28.6 cm (11.25 inch) rods (P23), and two 40 cm rods (Fig. 22).

**Fig. 22:**
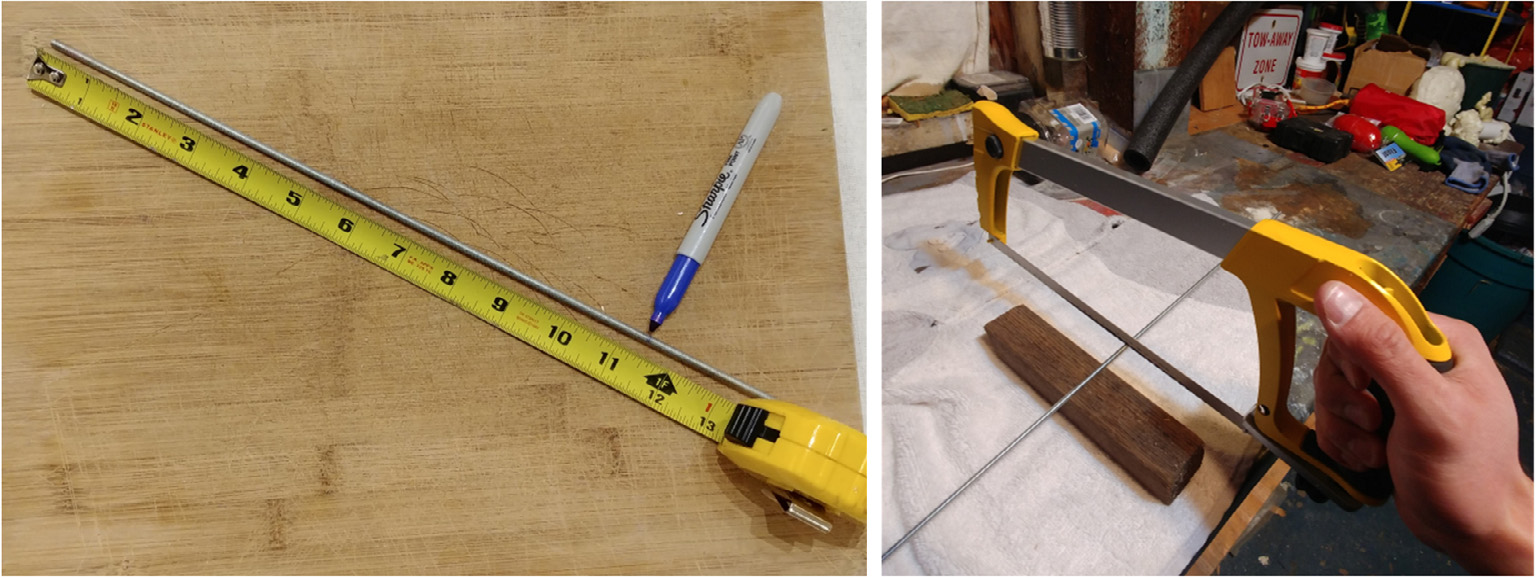
Cutting of the metal rods.
9. Attach all the rods to the connector-free disk using washers (P24), lock-washers (P26) and nuts (P25) on both sides. For one of the central rods, the washers, lock-washers and nuts should only be used on the external side of the disk, so it can be easily removed when installing the D-cell batteries. The longer 40 cm rods should be in the outside holes (depicted in green in Fig. 20) and the short ones should be in the central holes. Pass the red wires of the positive disk through the second wire hole, then slide the disk through the rods and stack it on top of the connector-free disk (Fig. 23).

**Fig. 23:**
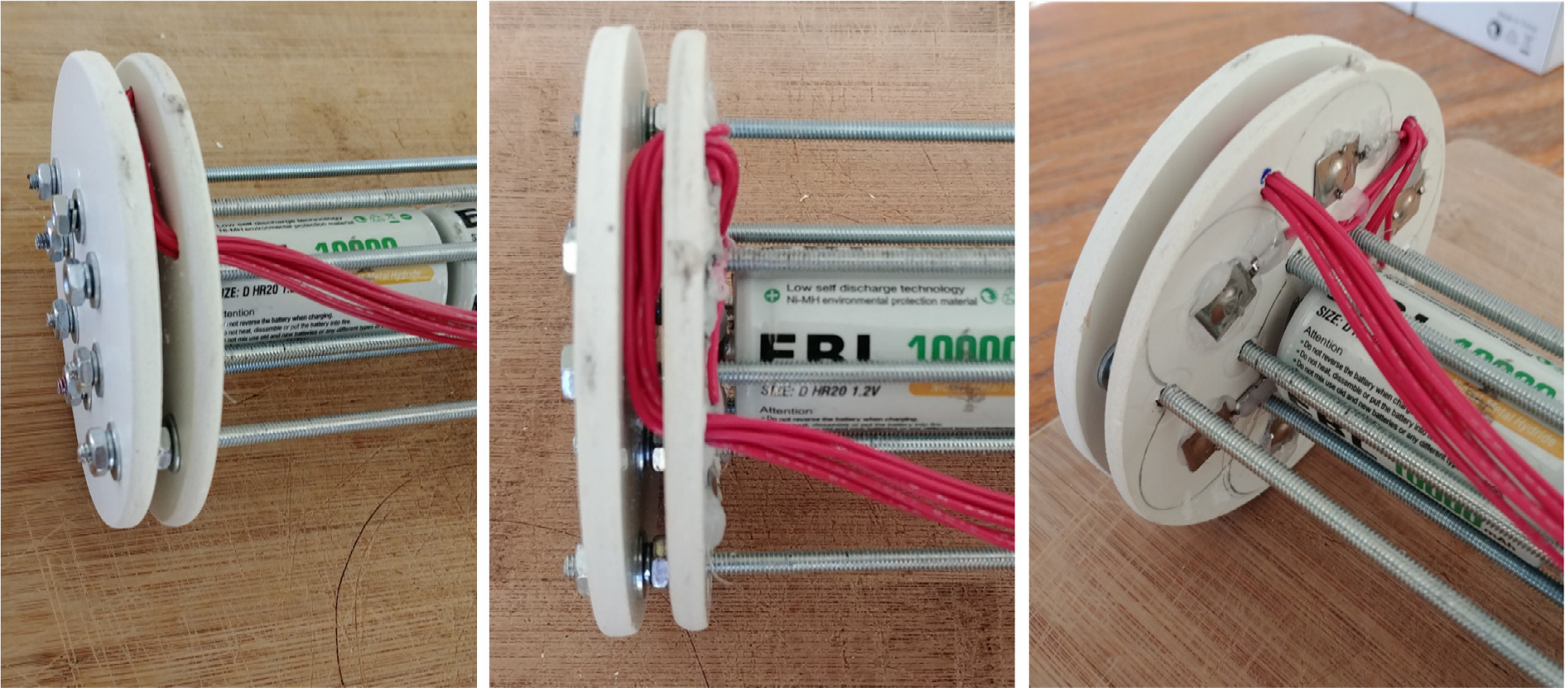
Assembly of the rods and discs.
10. Slide the negative disk through the rods with the spring connectors facing the plate connectors of the positive disk, and secure it to the rods using the washers (P24), lock-washers (P26) and nuts (P25) on both sides. As for the positive disk, one of the central rods should have washers, lock-washers and nuts on one side only to be able to remove the rod easily when installing the D-cell batteries. Use four D-cell batteries between the centrals rods to ensure the distance between the positive and negative disks is appropriate (i.e. long enough to fit four D-cell batteries, but short enough to maintain the D-cell batteries tightly in place). Finally, pass the red wires from the positive disk through the remaining wire hole of the negative disk (Fig. 24).

**Fig. 24:**
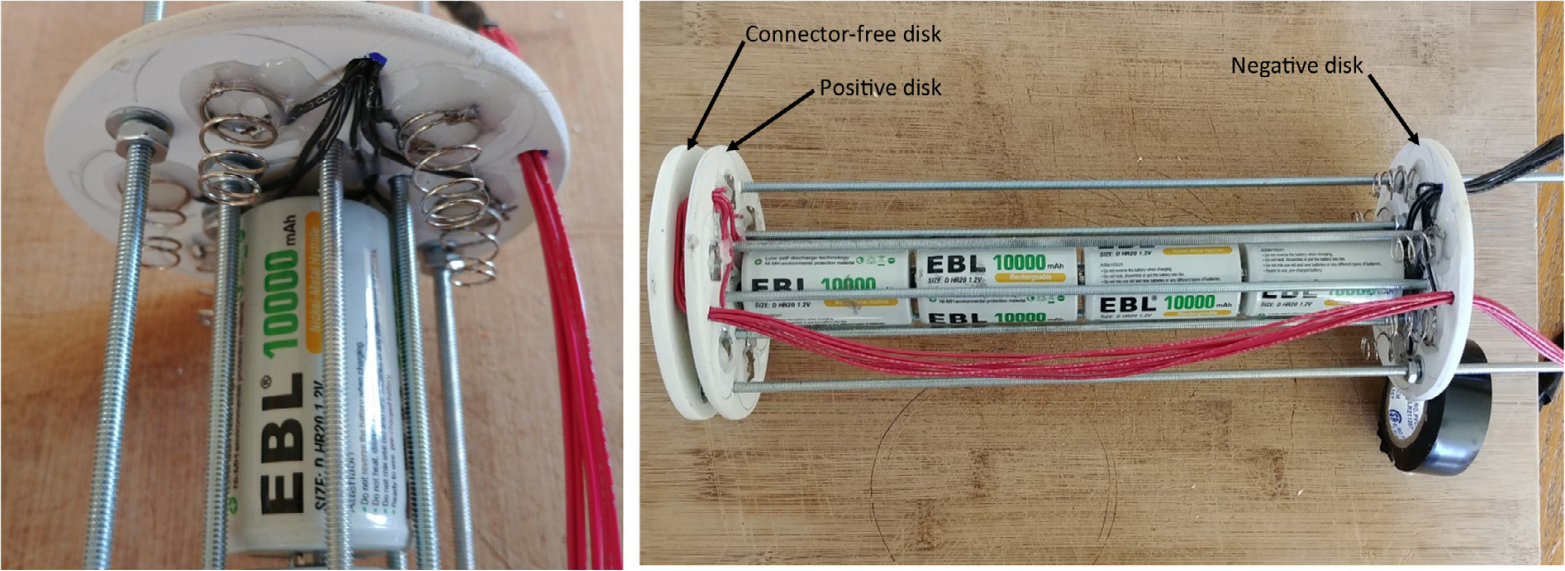
Assembly of the negative discs.
11. Solder the free end of the red and black wires to the Molex battery connector (P13, Fig. 25).

**Fig. 25:**
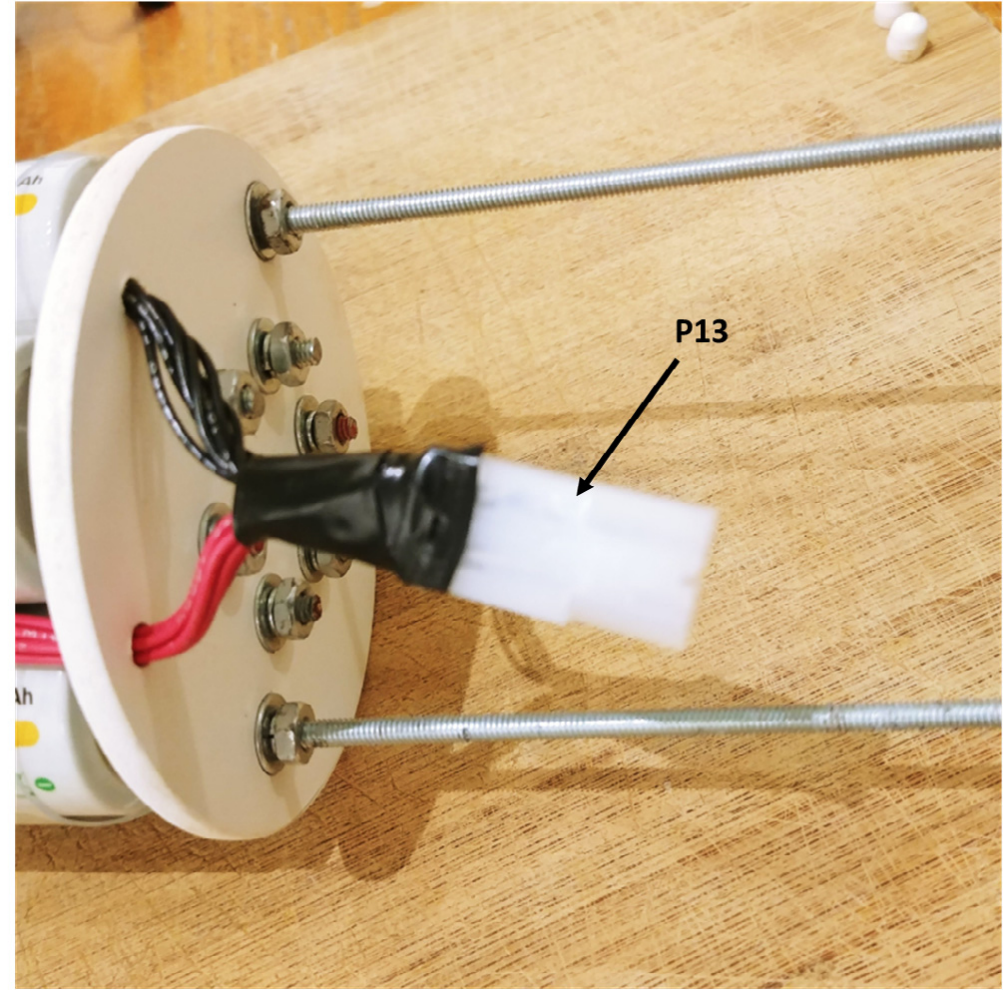
Soldering of the Molex connector.
12. Cut a small piece of metal hanger strap (P31), bend it as a finger handle and attach it to the outside of the connector-free disk with the washers (P24), lock-washers (P26) and nuts (P25, Fig. 26).

**Fig. 26:**
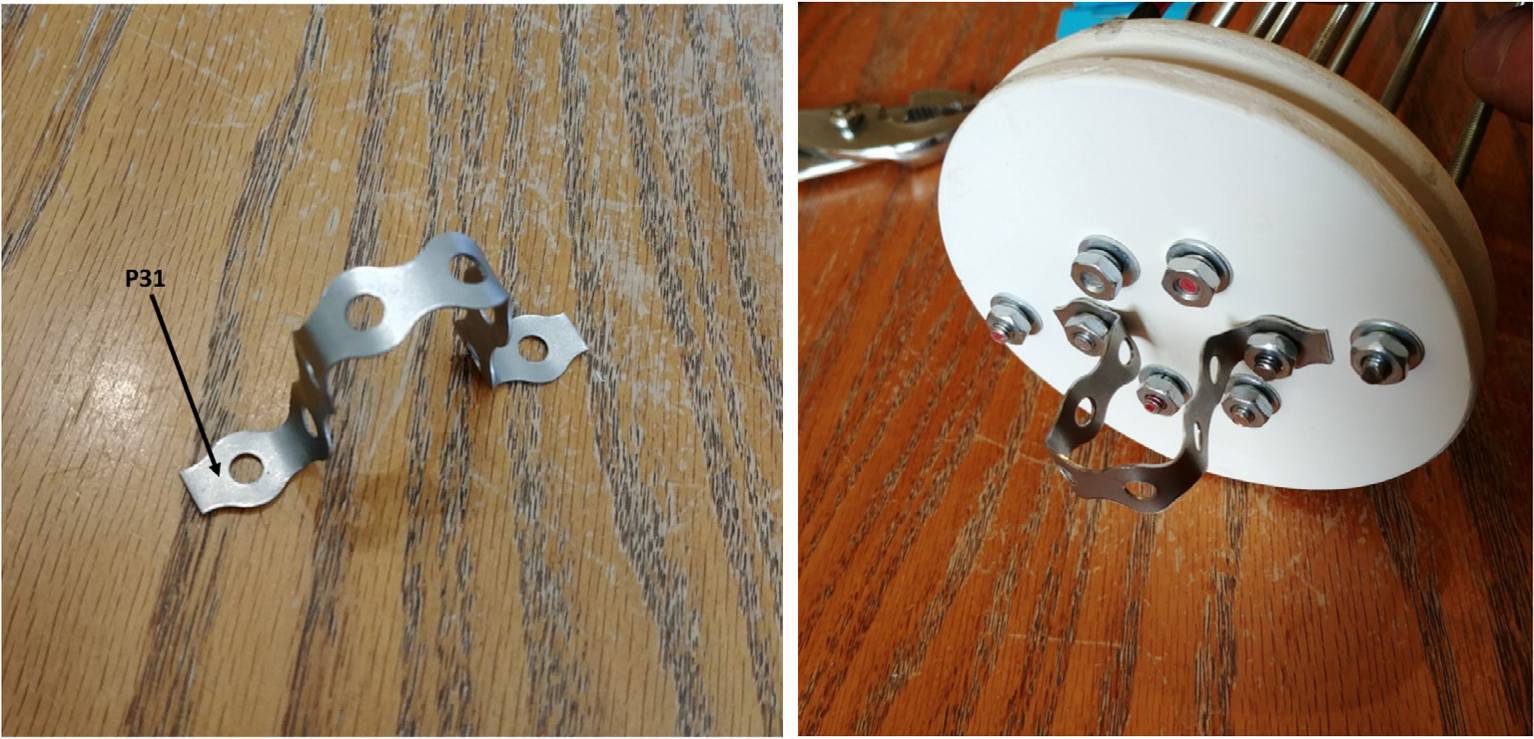
Attachment of the handle.
13. Slide the mounting plate with the electronics through the rods (Fig. 27).

**Fig. 27:**
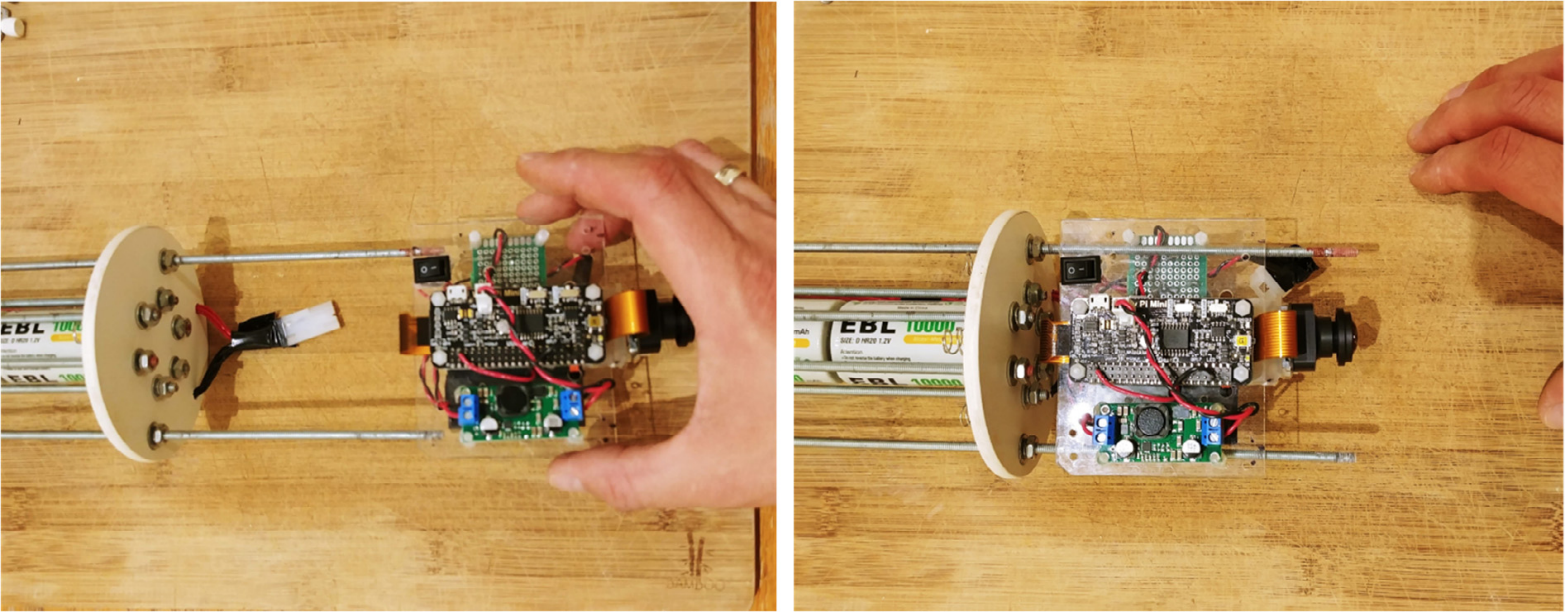
Assembly of the electronics with the internal frame.
14. Secure the mounting plate to the rods with four small cable ties (P32) and connect the board to the battery pack with the Molex battery connectors (P13, Fig. 28).

**Fig. 28:**
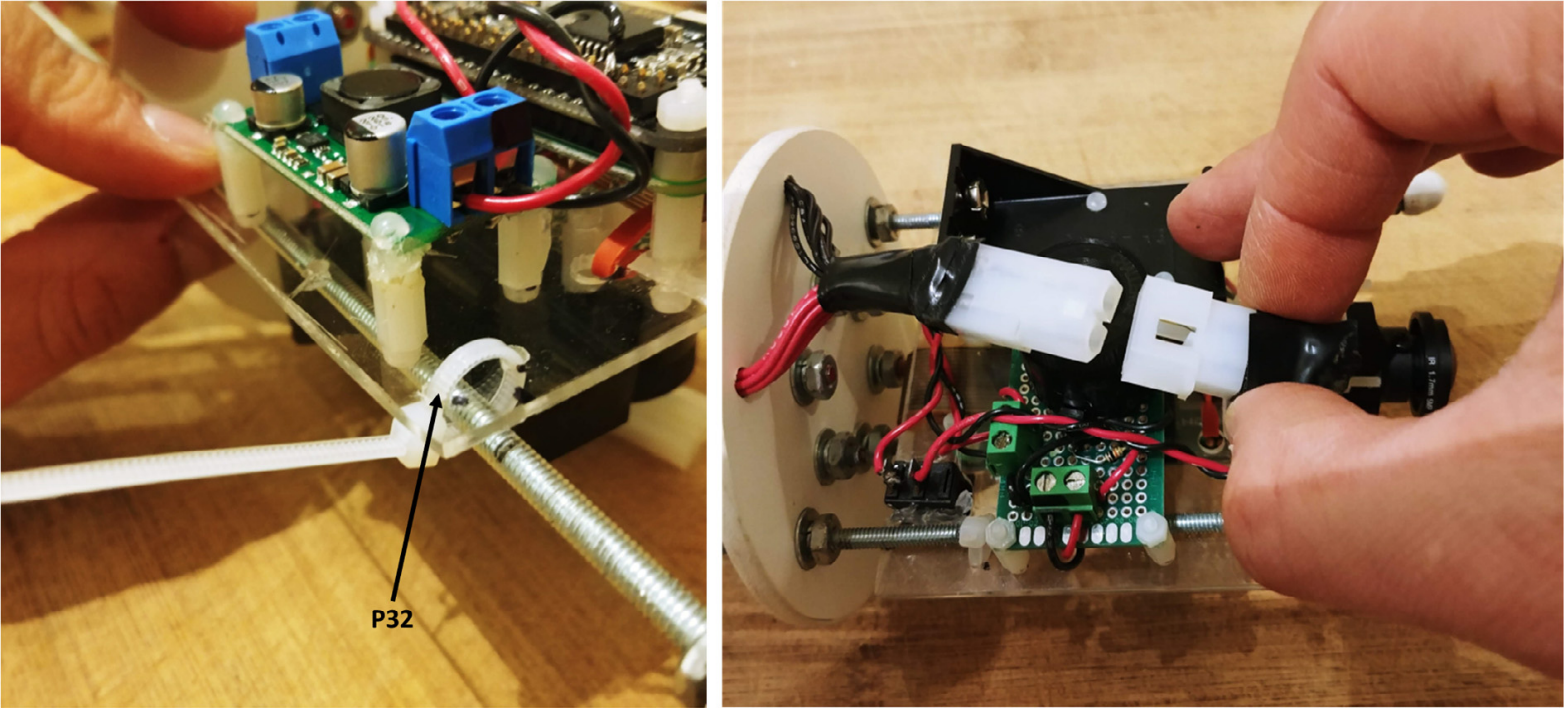
Connection of the electronics to the battery pack.
15. Add a nut (P25), lock-washer (P25), and cap nut (P27) to the end of the two long rods and cover the cap nut with a screw protector (P28, Fig. 29).

**Fig. 29:**
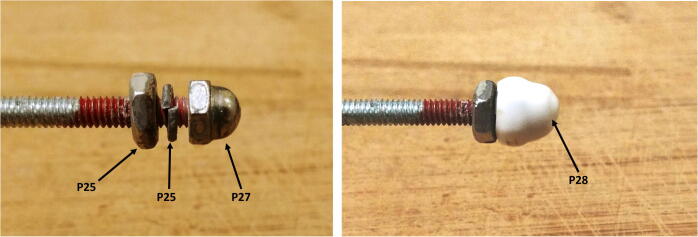
Assembly of the cap nut and screw protector.
16. The inside of the FishCam is now assembled (Fig. 30).

**Fig. 30:**
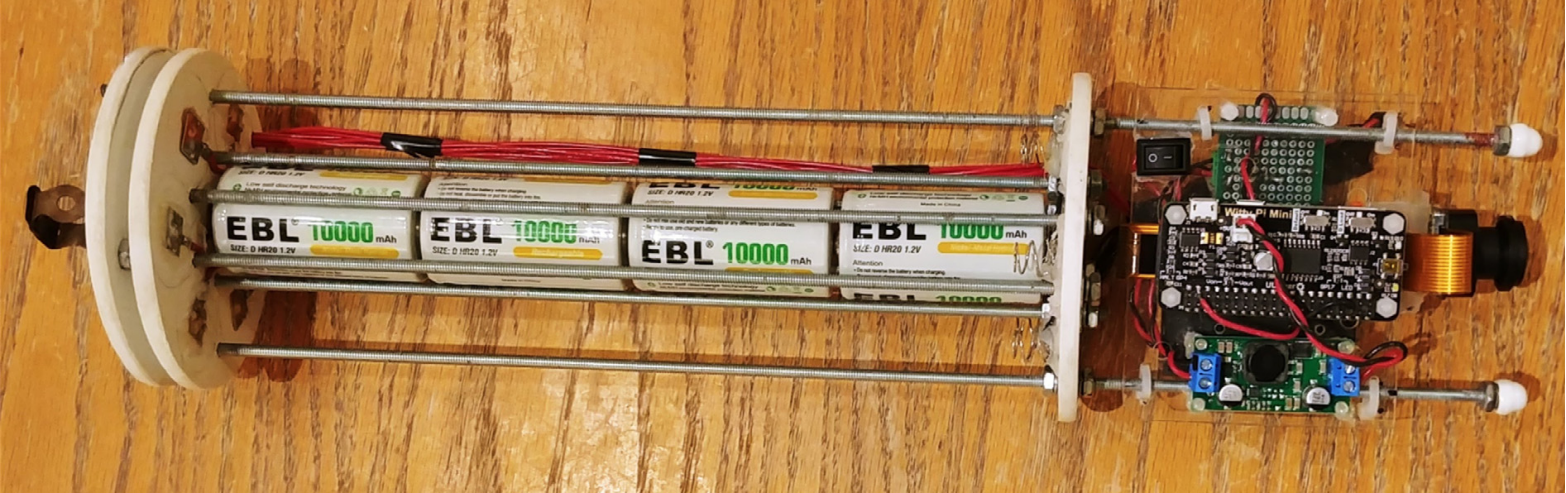
Inside of the Fishcam fully assembled.

### 5.3 Building the pressure housing and external attachment

All the instructions to build the custom PVC housing are in the supplementary material of Bergshoeff et al. [15]. The off-the-shelf 4” pressure housing can easily be ordered from the Blue Robotics website (https://bluerobotics.com/store/watertight-enclosures/4-series/wte4-asm-r1/).

The external attachment for the FishCam is assembled as follows.

1. Using a saw, cut an ABS 4” × 4” × 2” tee fitting (P35) in half, lengthwise (Fig. 31).

**Fig. 31:**
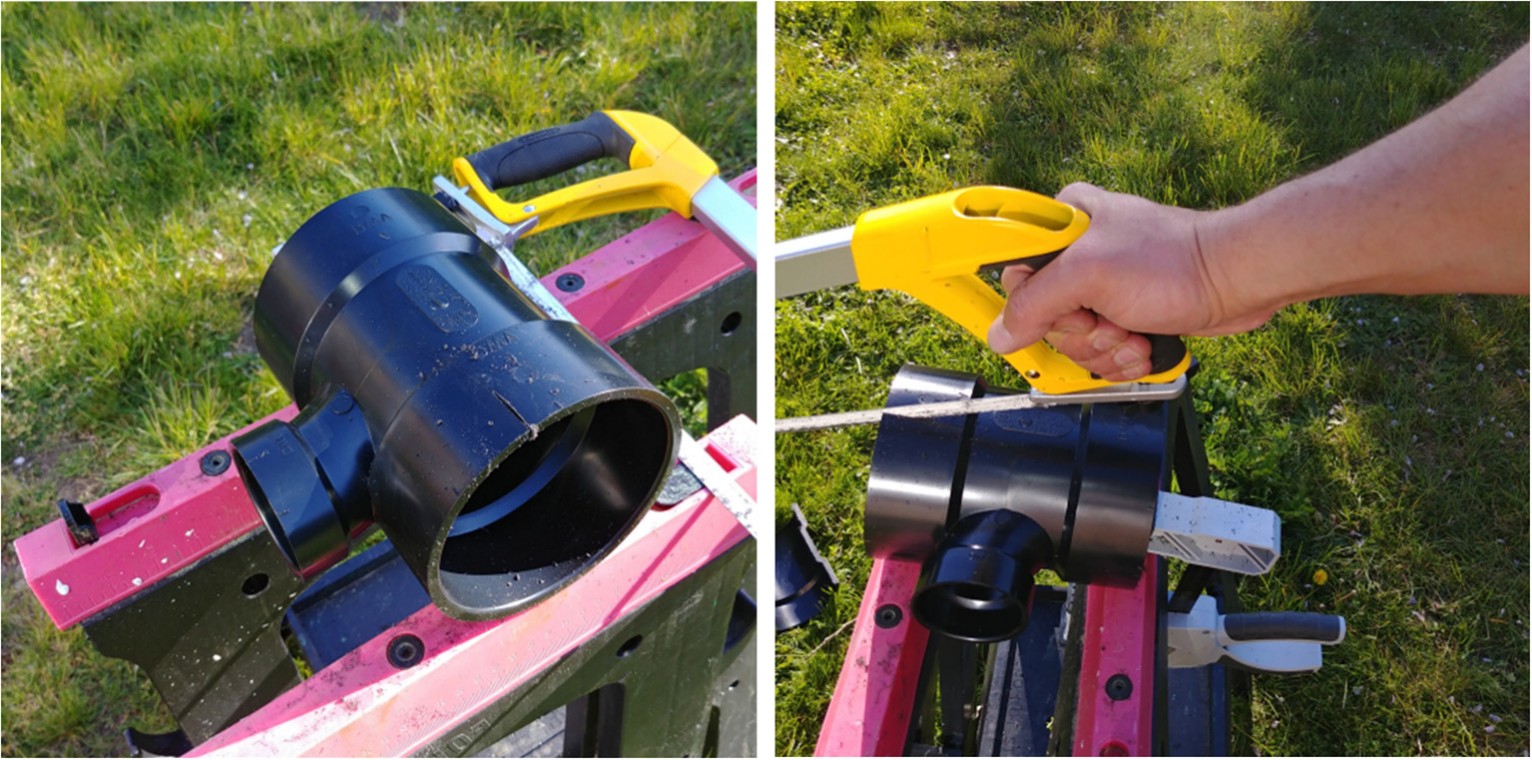
Cutting of the tee fitting.
2. Glue a 2” to 1–1/2” flush bushing fitting (P36) to the tee fitting using PVC cement (Fig. 32).

**Fig. 32:**
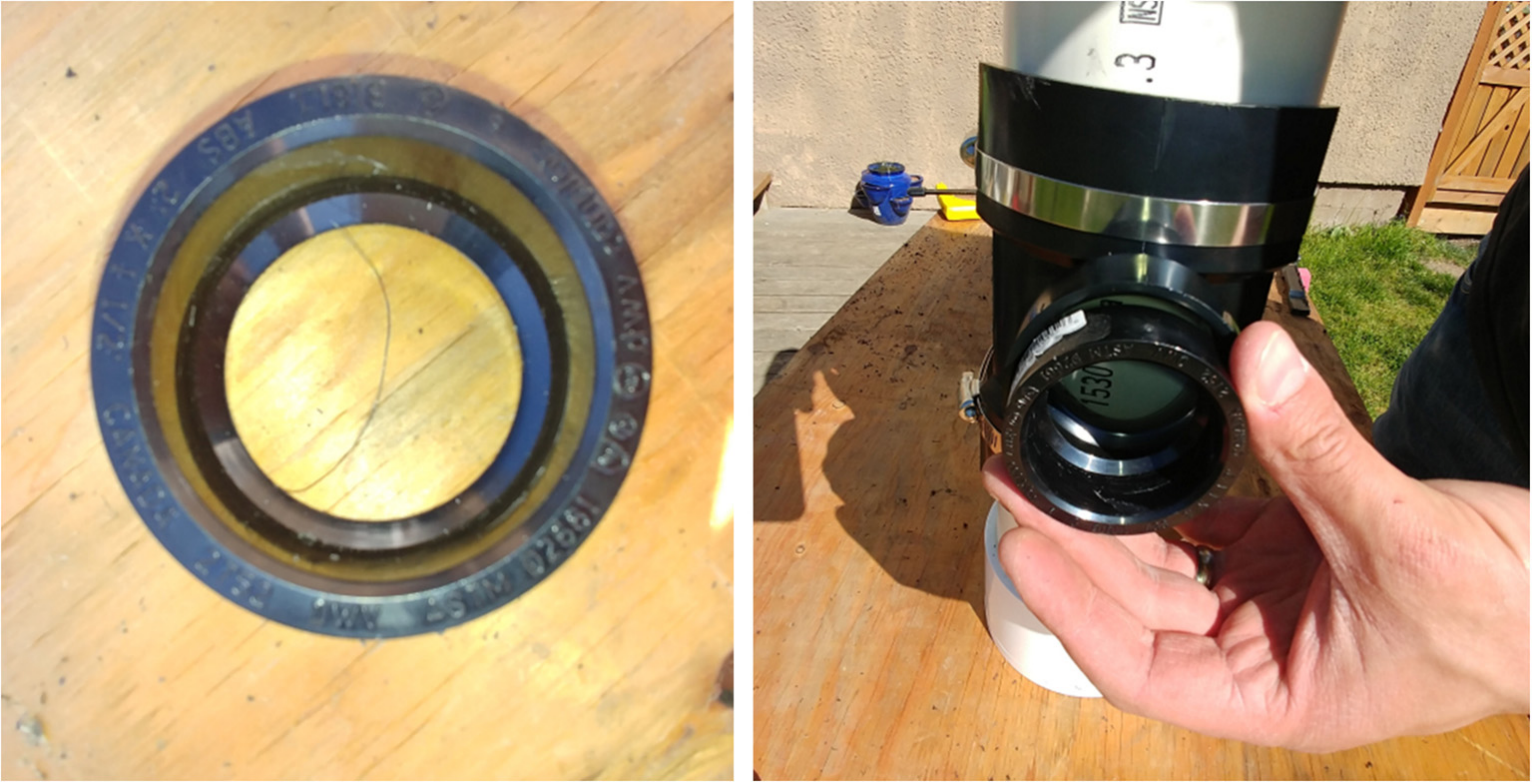
Installation of the bushing on the tee fitting.
3. (a) Cut a small piece of 1–1/2” PVC pipe (P38) and attach it to the PVC union fitting (P37) using PVC cement. (b) Attach the PVC union to the tee fitting using PVC cement (Fig. 33).

**Fig. 33:**
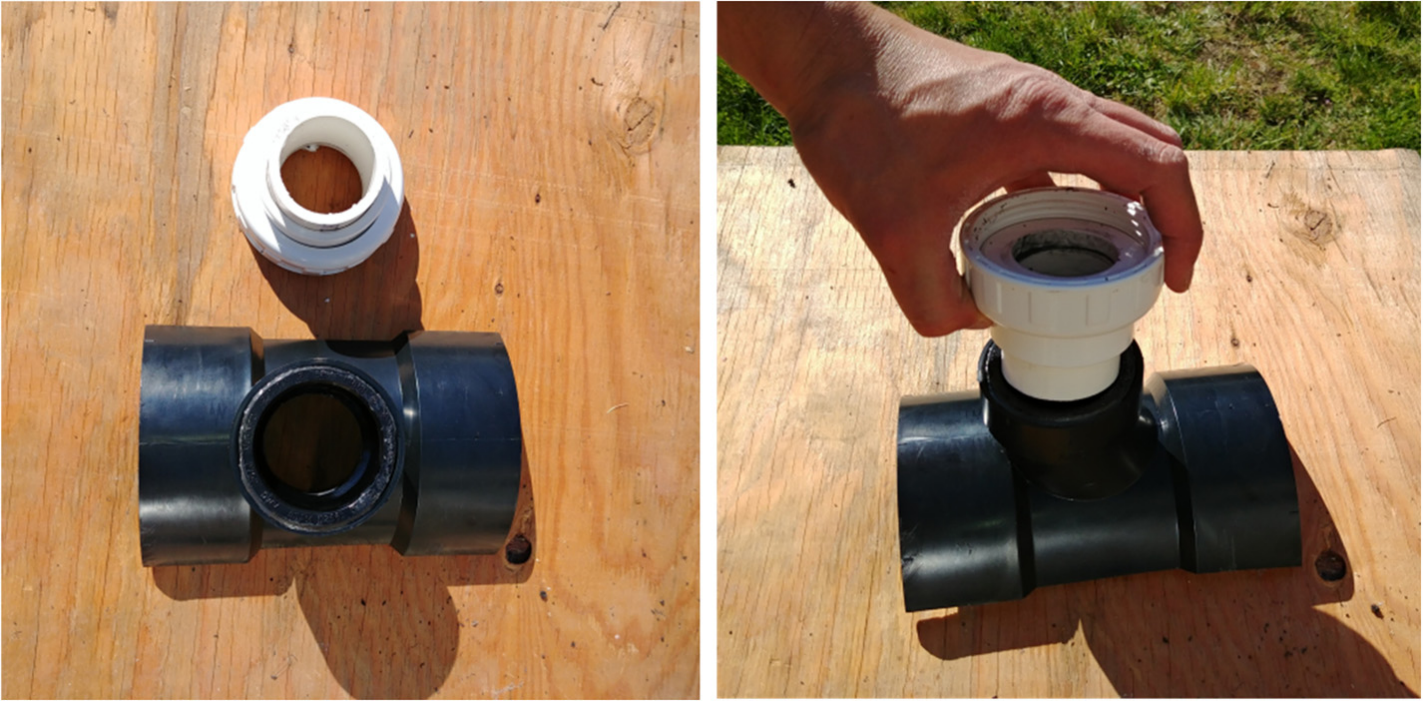
Installation of the PVC union fittings.
4. Repeat steps 1–3.
5. Place the two halves of tee fittings on either side of the pressure housing and secure with three adjustable stainless steel collars (P39, Fig. 34).

**Fig. 34:**
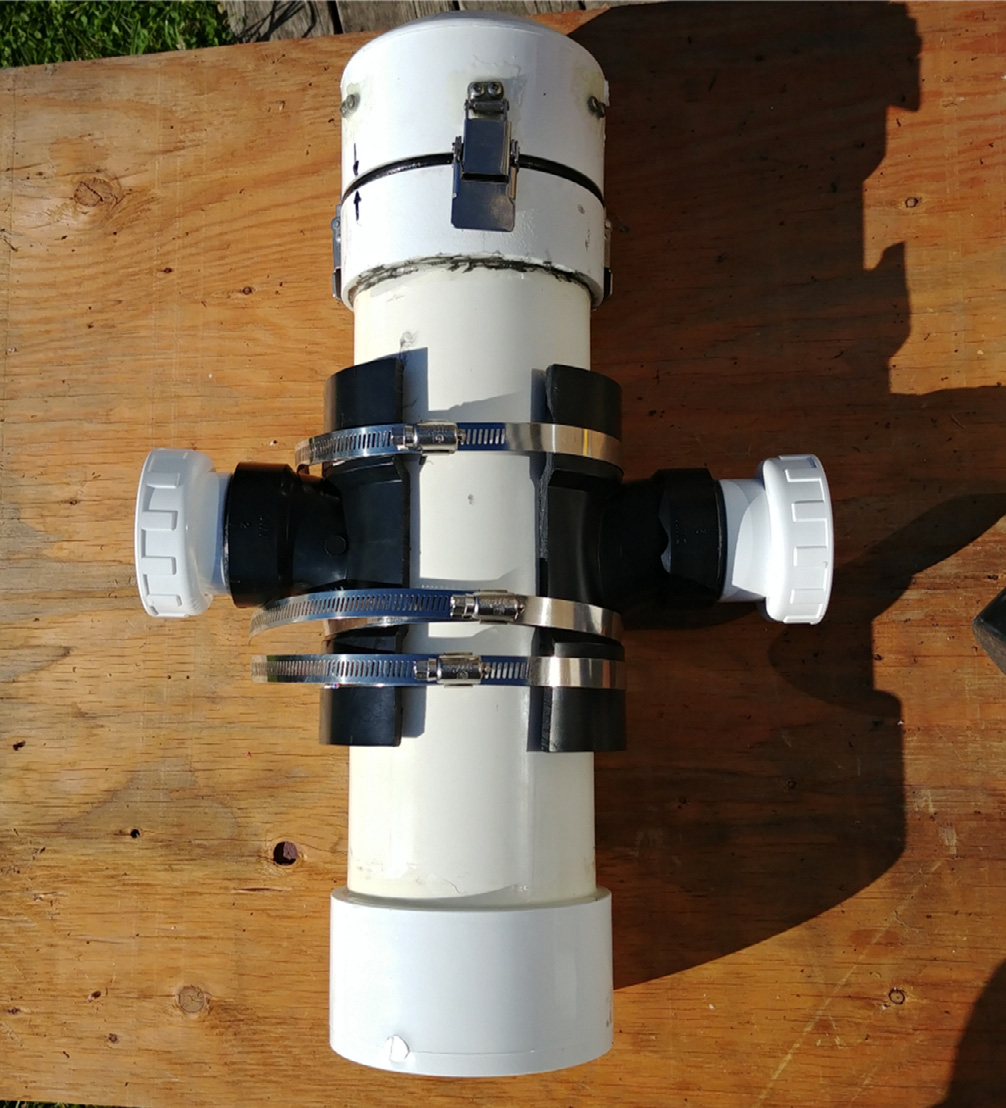
Installation of PVC attachments to the pressure housing of the Fishcam.

### 5.4 Installing the D-cell batteries

This section describes the steps to install the D-cell batteries in the FishCam.

1. Test with a voltmeter that all the D-cell batteries are properly charged.
2. Unscrew the end of one of the central rods (the one with no bolts on the inside of the negative disk) and slide it out. Insert the four D-cell batteries in the central battery stack. Slide the rod back and secure it with the bolts and nuts (Fig. 35).

**Fig. 35:**
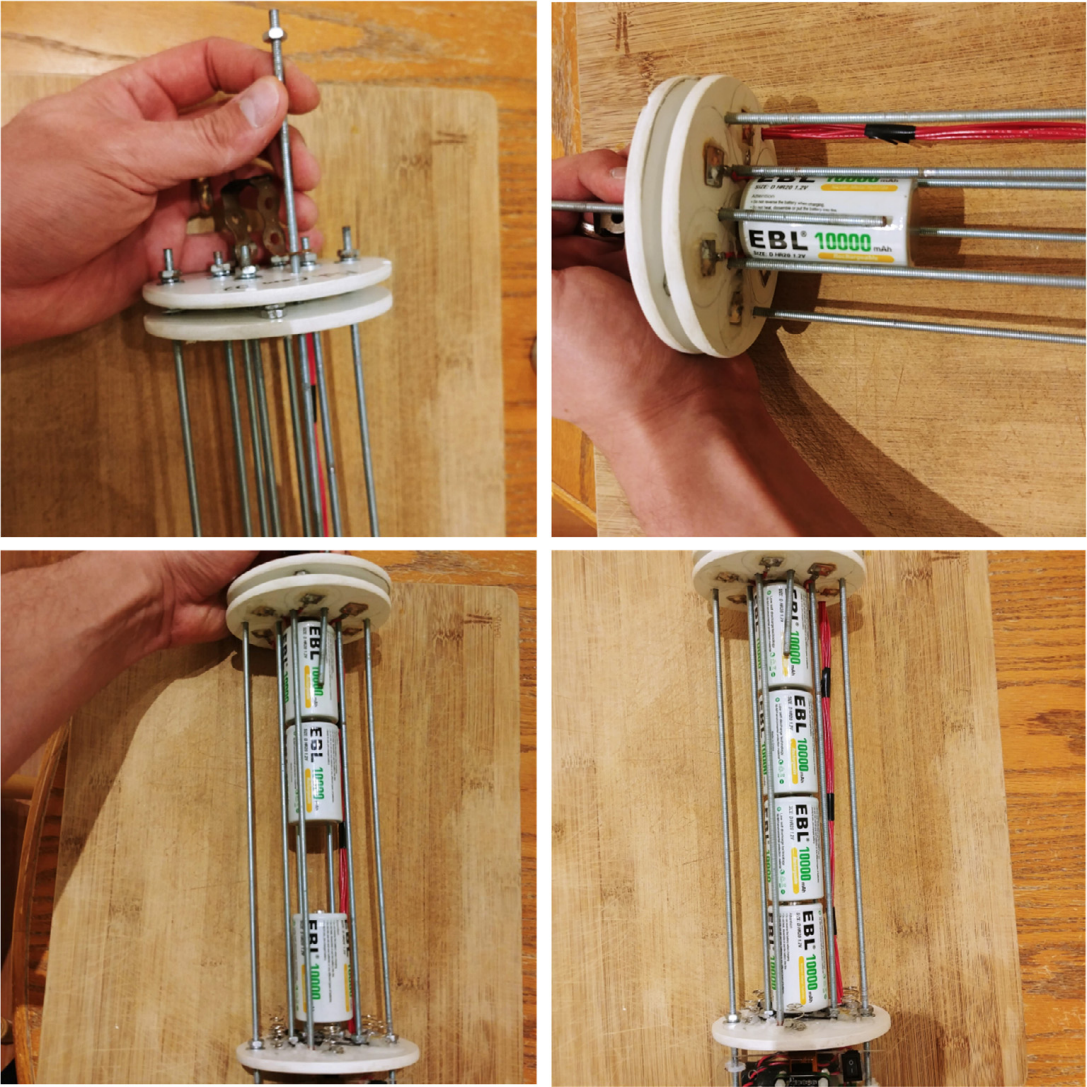
Installation of the D-cell batteries in the central stack.
3. Wrap the laminated plastic sheet (P29) around the rods and hold it in place with your hand.
4. Slide the plastic sheet down towards the positive-disk and insert the first row of 6 batteries.
5. Slide the plastic sheet slightly up and insert the second row of batteries. Repeat this process for the third and fourth rows of batteries.
6. Hold the plastic sheet tightly around the battery pack with your hand and use electrical tape to keep it in place. At this point the batteries should be well secured in place (Fig. 36).

**Fig. 36:**
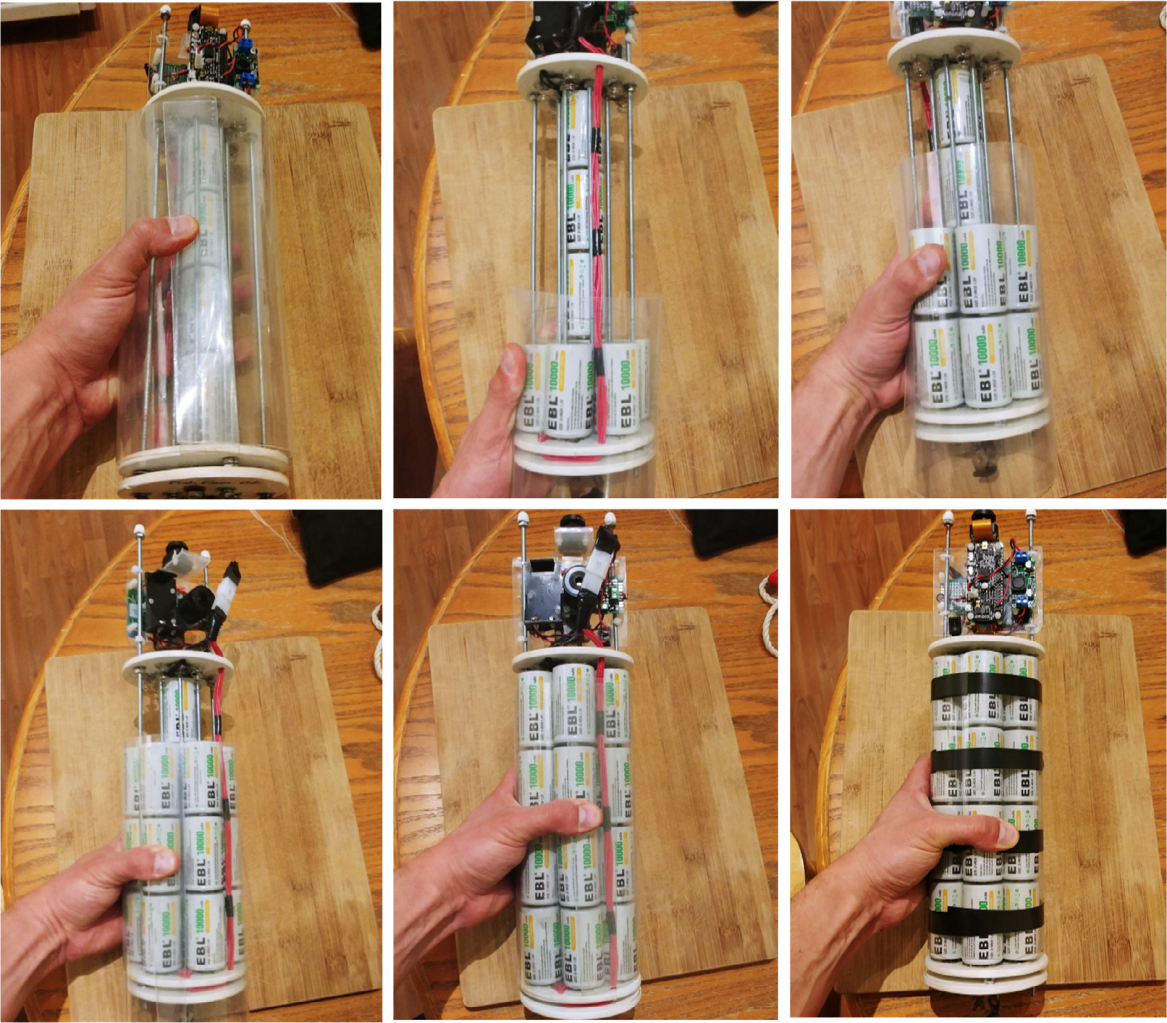
Installation of the rest of the D-cell batteries.
7. Measure the voltage of each 4-battery stack with a voltmeter. All stacks should have the same voltage value.

## 6 Operation instructions

This section describes how to set the FishCam to start recording when it is powered ON and how to adjust the video settings (frame rate, ISO, resolution, etc). For these instructions, you need to have the FishCam started and have access to the FishCam OS either directly via a mouse, keyboard and monitor, or remotely via an SSH connection (see Section 6.7.3).

### 6.1 Installing the software suite

Setting up the software requires both a computer and the FishCam. It is assumed here that the computer uses a recent version of the Microsoft Windows operating system (OS). Using a computer with a different OS is possible but is not documented here. It is also assumed that a Wifi connection with access to the internet is available. Steps 1–6 are performed on the Windows computer, and steps 7–14 are performed on the FishCam itself. For all these steps, it is recommended to power the FishCam via the power USB port of the Raspberry Pi using an external power supply (i.e. not the D-cell battery pack).

1. Take the microSD card (P9) and connect it to your computer.
2. Use free software such as *SD Card Formatter* to format the microSD card.
3. Download the image (.img) file *Raspbian Buster with Desktop* from the Raspberry Pi website (https://www.raspberrypi.org/downloads/raspbian/).
4. Copy the Raspbian image to the microSD card using free software such as *Win32 Disk Imager*.
5. Once Raspbian is installed on the microSD card it will appear on the computer as two separate drives *boot* and *rootfs*. On the *boot* drive, open the file *config.txt* with a text editor (e.g. Notepad) and add the line *dtoverlay* *=* *pi3-disable-bt* at the end of the file. Save and close the text editor. This step turns off the Bluetooth capabilities of the Raspberry Pi to save power. Ignore this step if you need to use the Bluetooth.
6. Eject the microSD card from the computer and insert it in the FishCam’s Raspberry Pi.
7. Connect a mouse, keyboard and monitor to the Raspberry Pi and turn the power on. This step will require a micro-USB to USB adapter, a USB hub, and a mini-HDMI to HDMI adapter. The Raspberry Pi will boot and the graphical interface of Raspbian will appear on the monitor.
8. Upon the first start, a window will appear with instructions to configure the OS. Follow all the instructions to set up the country, password, wifi connection, and updates.
9. If not already done, connect to your Wifi network using the Wifi icon at the top right of the screen.
10. Open a terminal window and type the following commands:
  (a) Ensure the OS is up to date:
    • **sudo apt-get update**
    • **sudo apt-get upgrade**
  (b) Install Python 3:
    • **sudo apt-get install python3**
  (c) Install the python picamera library:
    • **sudo apt-get install python3-picamera**
  (d) Install the python GPIO library:
    • **sudo apt-get install python3-rpi.gpio**
  (e) Install the crontab job scheduling tool:
    • **sudo apt-get install cron**
  (f) Install Git to download the FishCam scripts from GitHub:
    • **sudo apt-get install git**
  (g) Download the FishCam scripts from GitHub:
    • **cd/home/pi/Desktop/**
    • **git clone https://github.com/xaviermouy/FishCam.git.**(notice the **“. ”** at the end)
    • A folder named “FishCam” should now be on the Desktop and have the scripts to run the FishCam.
  (h) Install the WittyPi software:
    • **wget http://www.uugear.com/repo/WittyPi2/installWittyPi.sh**
    • **sudo sh installWittyPi.sh**
    • When prompted, type **y** to remove *fake-hwclock*, and **n** to not install Qt5
11. Restart the Raspberry Pi to apply all the changes.
12. Once Raspbian has restarted, open a terminal window and type **sudo raspi-config** to configure the Raspberry Pi:
  (a) If not done already, change your user password in the menu **Change User Password**.
  (b) In the menu **Network Options** select **Hostname**, and change it to **fishcam01**. Other hostnames can be chosen, but it has to be explicit enough to be easily identifiable on a network.
  (c) In the menu **Change Boot Options** and **Desktop/CLI**, select **Desktop Auto Login**.
  (d) To set FishCam time to UTC, in the menu **Localisation Options**, select **Change Time Zone**, then **None**, and **GMT**.
  (e) In the menu **Interfacing Options** set the **Camera (CSI camera interface)** and **SSH connection** to **enabled**.
  (f) Ensure the entire microSD card is used by selecting **Expand Filesystem** in the menu **Advanced Options**.
  (g) Select **Finish** to exit raspi-config.
13. At this point all the necessary software are installed.
14. Optional: shut down the Raspberry Pi, take the microSD card out and connect it to your computer. Use free software such as *Win32 Disk Imager* to take an image of the microSD card with all the software installed. The image file created may be used to set up another FishCam without having to go through all the installation steps described above.

### 6.2 Automatic start of the recordings

The crontab job scheduler is used to start acquiring video when the FishCam is powered ON (i.e. rebooted). Open a terminal window and type the following commands:

1. Edit the job schedule by typing: **crontab -e**
2. The first time you run crontab you will be prompted to select an editor. Choose Nano by pressing Enter.
3. Once the schedule is open in Nano:
  (a) Scroll down with the down-arrow key to the bottom of the document and type
    **@ reboot sh/home/pi/Desktop/FishCam/script/camStartup.sh** &
  (b) Save the changes by pressing CTRL  + O, then press Enter to confirm.
  (c) Exit Nano by pressing CTRL  + X.
4. Verify that the schedule has been saved:
  **crontab -l**
5. Close terminal

### 6.3 FishCam ID

If you are using several FishCams, it may be useful to assign a unique ID to each of them. This ID will be used at the beginning of the filename of each video being recorded. To modify the FishCam ID:

1. Go to the folder **/home/pi/Desktop/FishCam/script/**
2. Open the file **FishCamID.config** with a text editor.
3. Type the FishCam ID, save, and close the text editor. By default FishCam ID is set to *FishCam01*.

### 6.4 Camera settings

All the camera settings are defined in the python script **captureVideo.py** located in the folder **/home/pi/Desktop/FishCam/script/**. To change the settings, open the script **captureVideo.py** with a text editor and adjust the parameters defined in the function *initVideoSettings()*. For more information about the different parameters, refer to the documentation of the *picamera* library (https://picamera.readthedocs.io).

**Table:** 

def initVideoSettings():	
videoSettings = {	
’duration’: 300,	# files duration in sec
’resolution’: (1600,1200),	
’frameRate’: 10,	# frame rate fps
’quality’: 20,	# 1 = best quality, 20 - ok, 30 poorer quality
’format’: ’h264’,	# ’h264’, ’mjpeg’
’exposure’: ’night’,	# ’auto’, ’night’,’backlight’
’AWB’: ’auto’,	# ’auto’, ’cloudy’, ’sunlight’
sharpness’: 0,	# integer between −100 and 100, auto: 0
’contrast’: 0,	# integer between −100 and 100, auto: 0
’brightness’: 50,	# integer between 0 and 100, auto: 0
’saturation’: 0,	# integer between −100 and 100, auto: 0
’ISO’: 400,	# low sensitivity: 100, high sensitivity: 400,800, auto: 0
’vflip’: False	
}	
return videoSettings	

### 6.5 Configuring the duty cycles

The duty cycles of the FishCam are configured using the software provided with the Witty Pi and installed in Section 6.1. In this section, it is assumed that the FishCam is connected to the internet via Wifi.

1. Create a Witty Pi schedule script (i.e. .wpi file) to define the ON and OFF sequence desired and save it in the folder **/home/pi/WittyPi/schedules/**. The name of the.wpi file should be explicit and self-explanatory (e.g. *schedule*_*fishcam*_*fall*_*UTC.wpi*). Examples of schedule scripts can be found in the folder **/home/pi/Desktop/FishCam/wittypi**_**schedules/**. For more information on how to define ON/OFF sequences, refer to the Witty Pi user manual.
2. Open a terminal window:
  (a) Ensure the clock of the FishCam is at the correct time by typing the command **date**. If the date and time displayed are not correct, make sure that the FishCam is connected to the internet. The time should be updated automatically once an internet connection is established.
  (b) Start the Witty Pi software:
    **sudo sh/home/pi/WittyPi/wittyPi.sh**
  (c) Select menu item **3: Synchronize time**.
  (d) Ensure the system clock and the RTC clock at the top of the menu options show the same time. If not, re-run the time synchronization.
  (e) Select menu item **6: Choose schedule script**, and choose the name of the schedule script you created at the first step.
  (f) Select menu item **8: Exit.**
3. The duty cycles are now activated and the FishCam will start to turn ON and OFF based on the schedule defined.

**Note: The witty Pi mini cannot remember the time (RTC) without power for more than 17 h. Consequently, the time synchronization and schedules should be defined no more than 17** **h before the deployment of the FishCam**.

### 6.6 Configuring the buzzer

The beeping sequence of the buzzer can be defined by editing the file **/home/pi/Desktop/FishCam/script/runBuzzer.py** with a text editor. The five parameters below can be adjusted:

• **beep**_**dur**_**sec**: Duration of a single beep in seconds.
• **beep**_**gap**_**sec**: Duration of the silence between beeps in seconds.
• **beep**_**number**: Number of beeps in a sequence.
• **number**_**beep**_**sequences**: Number of sequences.
• **gap**_**btw**_**sequences**_**sec**: Duration of the silence between sequences.

**Fig. 37:**
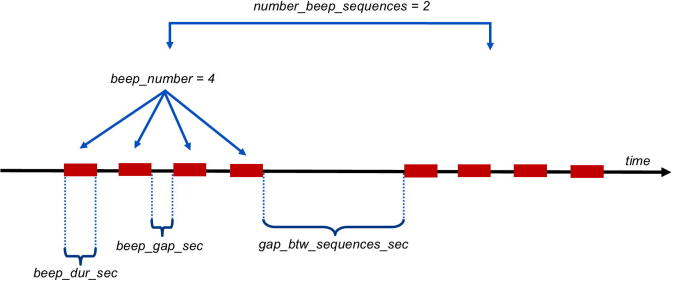
Illustration of the parameters defining the beeping sequences of the buzzer.

Fig. 37 illustrates the different parameters that are used to define the beeping sequences.

### 6.7 Accessing the FishCam wirelessly

While connecting the FishCam to a keyboard, mouse and monitor is needed at the start to install the required software, it is not a necessity thereafter. Changing the configuration and operating the FishCam can be done wirelessly via a mobile device or a regular computer. The easiest way to do this is to create a local network by turning a cell phone into a Wifi hotspot. This works equally well on Android or Apple devices, but the steps to follow using the Android 9 OS on a LG G6 phone are given here.

#### 6.7.1 Creating a Wi-fi hotspot

1. On your Android mobile device go to the **System** menu, then **Network** & **internet**, **Tethering**, and **Wi-fi hotspot**.
2. Choose **Set up Wi-fi hotspot** and enter the Wi-fi name and password of your choice then save. In the example below, the name of the Wi-fi hotspot is *xraspi* (Fig. 38).

**Fig. 38:**
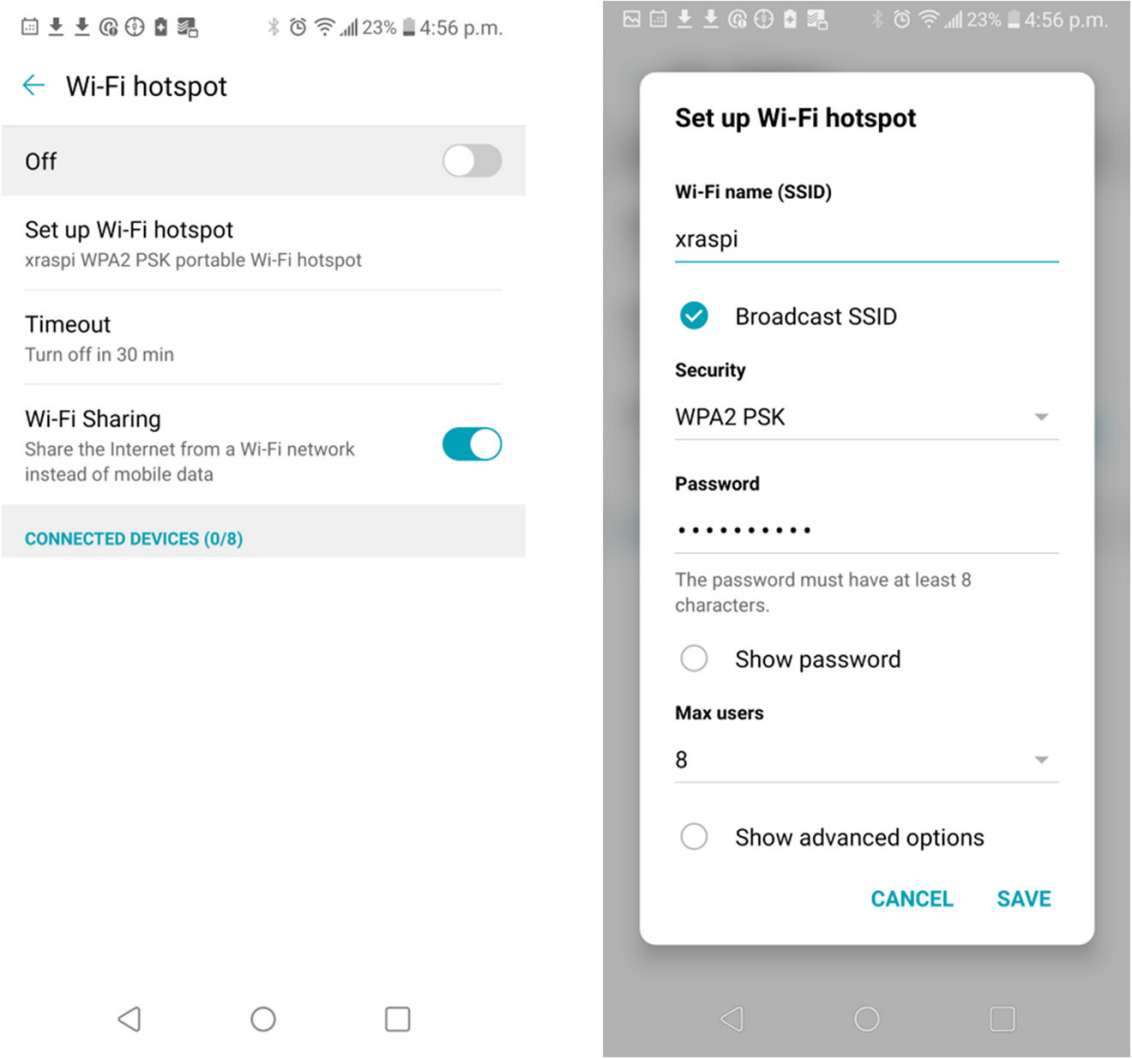
Setting up a Wi-fi hotspot.
3. Turn ON **Wi-fi Sharing** and finally turn ON the Wi-fi hotspot on your phone.
4. Connect the FishCam to a keyboard, mouse and monitor and turn it on. This will be the last time you will need to connect the FishCam to a monitor.
5. Once started, click on the Wi-fi icon at the top right of the screen, select your Wi-fi hotspot (here xraspi) and enter the corresponding password (the one set in step 2). Now the FishCam should connect to this network automatically if it is available. To ensure this is actually the case, before taking off the keyboard, mouse and monitor, you can reboot the FishCam and confirm that it connects automatically to the Wi-fi hotspot.
6. At this point all the keyboard, mouse, and monitor can be disconnected.

#### 6.7.2 Controlling the FishCam from a mobile device

Several phone applications exist to control the Raspberry Pi. Here we provide instructions for the free application RaspController by Ettore Gallina.

1. Turn ON the Wi-fi hotspot on your cell phone.
2. Turn ON the FishCam.
3. On you cell phone go in the menu **System**, then **Network** & **internet**, **Tethering**, and **Wi-fi hotspot**.
4. After a moment the FishCam should appear in the “Connected Devices” section. Note the FishCam’s IP address (in the example in Fig. 39, the IP address is 192.168.43.95)

**Fig. 39:**
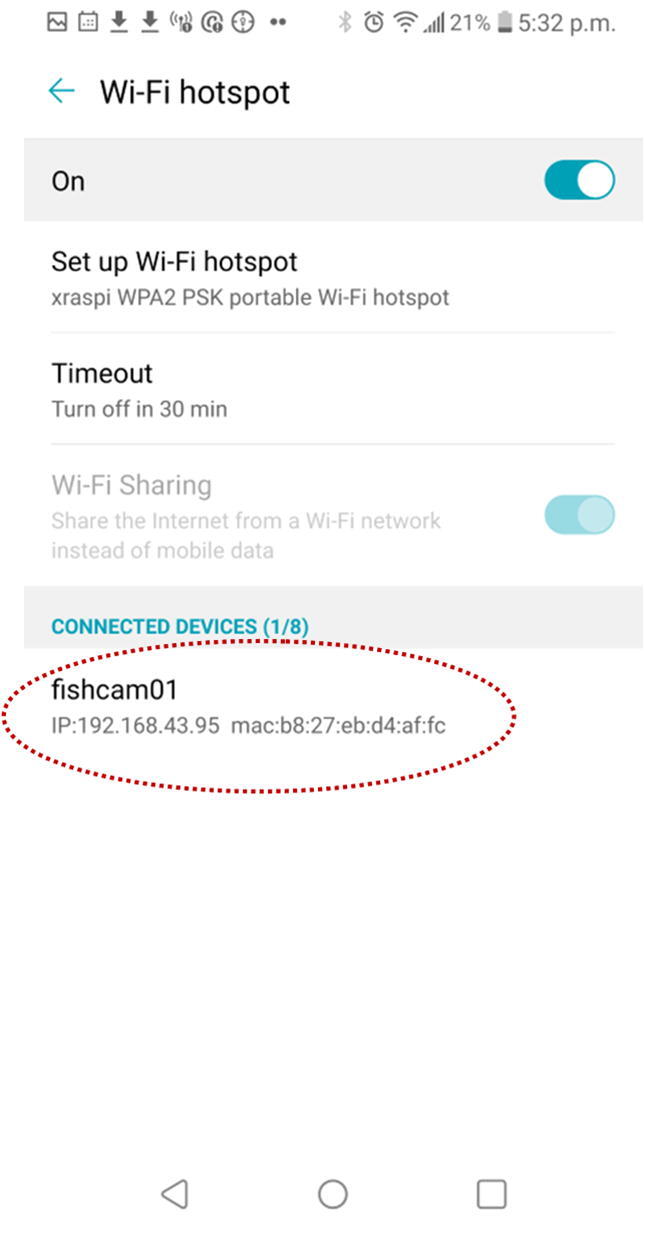
FishCam connected to the wi-fi hotspot.
5. Start the application RaspController on your phone, add a device (+ sign at the top right), then enter the IP address and password (the one defined in Section 6.1) of the FishCam and save.
6. Once connected to the FishCam, you can browse through the folders and files via the **File Manager** menu, open a terminal window via the menu **Shell SSH**, and monitor the FishCam resources via the menu **Cpu, RAM, Disk monitoring** (Fig. 40).

**Fig. 40:**
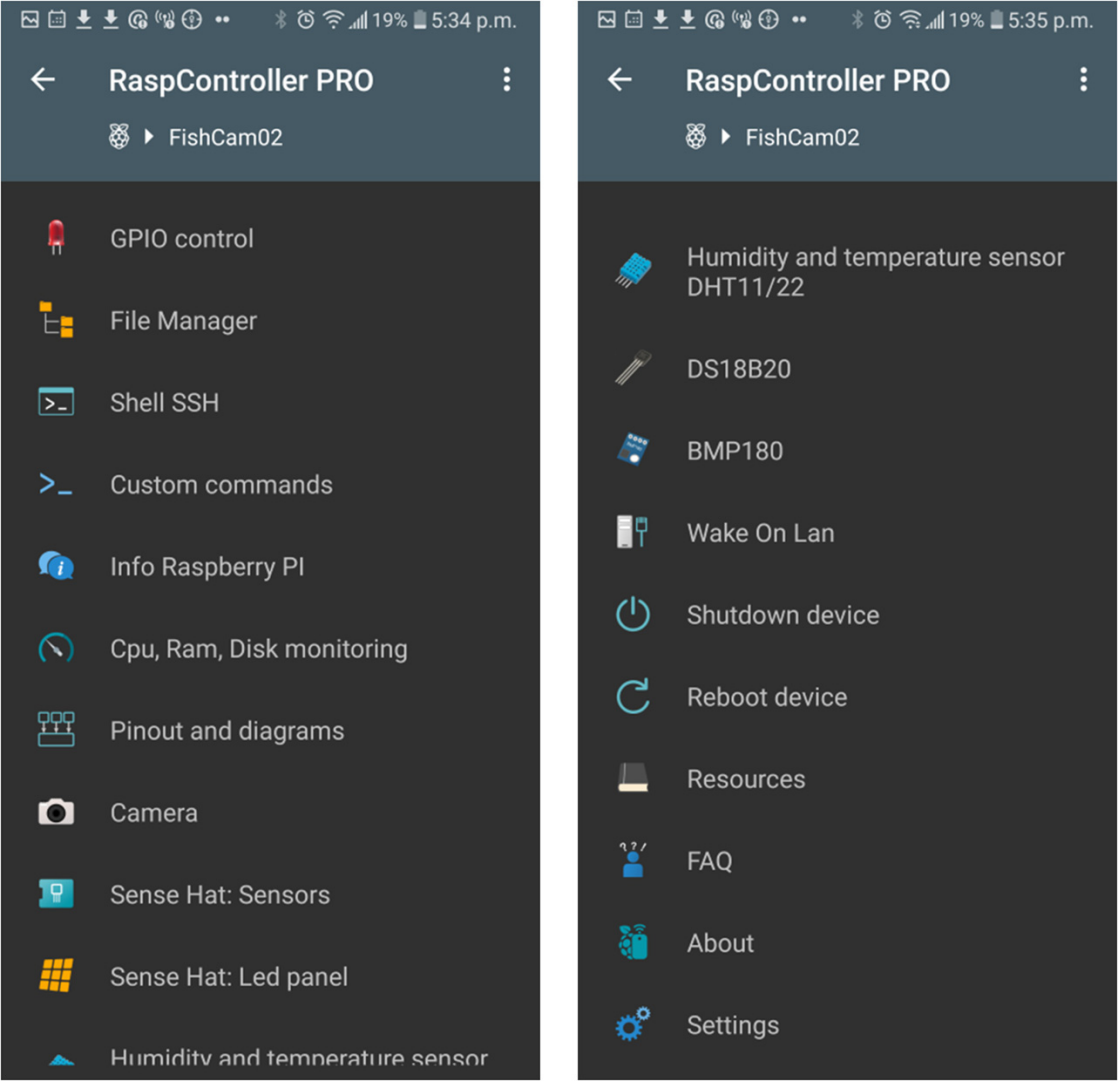
RaspController’s interface to control and monitor the FishCam.
7. Through the File Manager, it is possible to monitor that everything is working properly by verifying that video files are being recorded in the folder/home/pi/Desktop/FishCam/data/, verify their size, and read the logs to ensure all processes started properly and that there are no errors (Fig. 41).

**Fig. 41:**
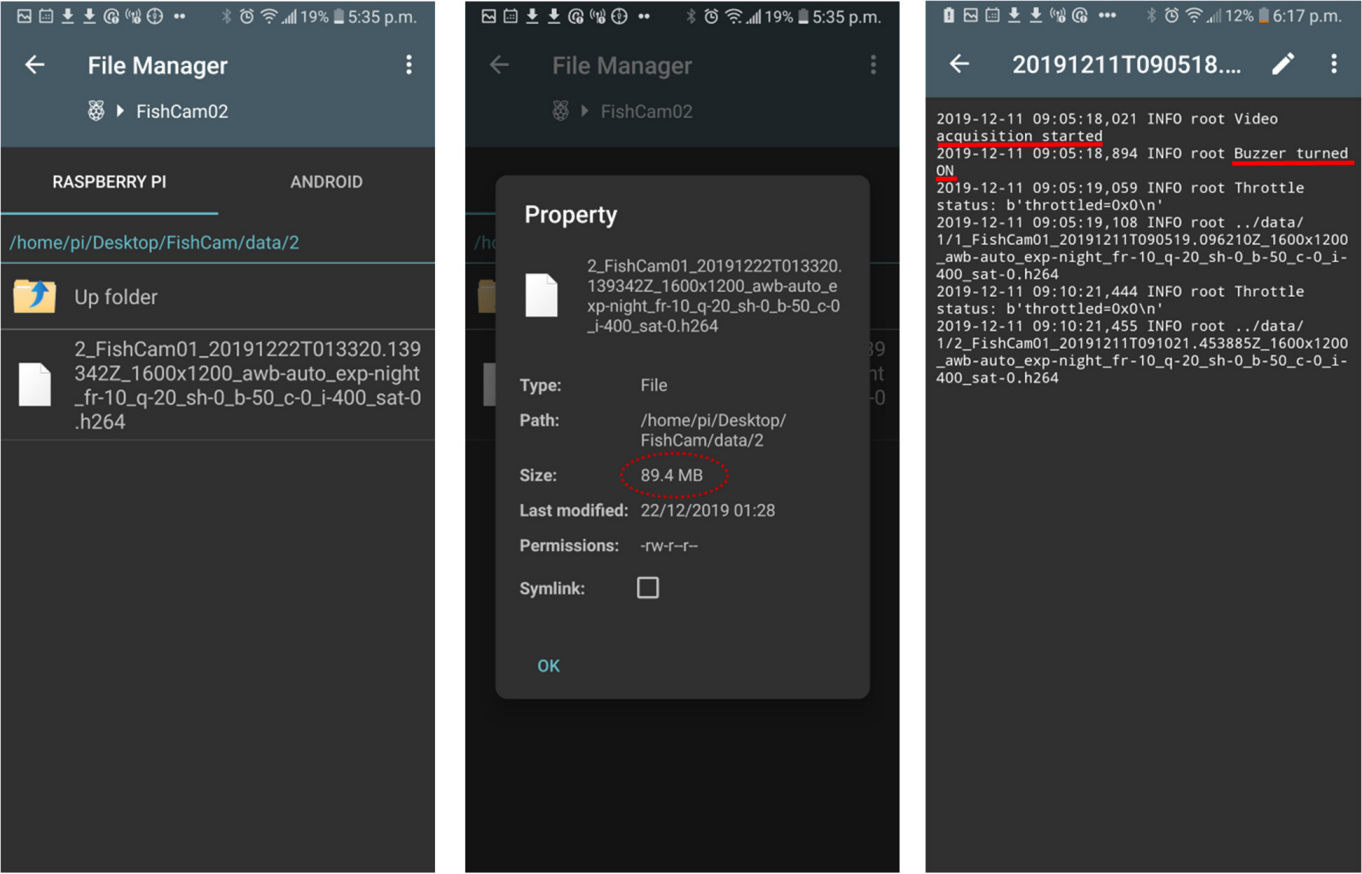
Monitoring the status of the data acquisition.

#### 6.7.3 Controlling the FishCam from a computer

The FishCam can also be accessed wirelessly from a computer. While there are several ways to do this, here we provide instructions using a Wi-fi hotspot (Section 6.7.1).

1. Turn ON the Wi-fi hotspot on your cell phone.
2. Connect to the Wi-fi hotspot on your computer.
3. Turn ON the FishCam.
4. On your cell phone go into the **System** menu, then **Network** & **internet**, **Tethering**, and **Wi-fi hotspot**.
5. After a moment the FishCam should appear in the “Connected Devices” section. Note the FishCam’s IP address.
6. On your computer, use a SFTP client such as FileZilla to connect to the FishCam and browse through the folders and files. You will need to connect using the FishCam’s IP address, username (i.e. “pi”), password (the one defined in Section 6.1) and using the port 22.
7. On your computer, use an SSH client such as Putty to access the terminal console of the FishCam. You will also need to connect using the FishCam’s IP address, username, password, use port 22 and an SSH connection type.

### 6.8 Downloading data from the FishCam

The fastest way to download the data from the FishCam is to take the microSD card out, connect it to a computer, and copy the **data** and **logs** folders to the computer. It is also possible to connect wirelessly to the FishCam from a computer and transfer the data via SFTP client software (see Section 6.7.3). It is recommended to download both the **data** folder, which contains all the video files, and the **logs** folder, which contains information about the start time of the buzzer sequences and any errors that may have occurred during the data acquisition.

**Data folder:**

/home/pi/Desktop/FishCam/data/

**Logs folder:**

/home/pi/Desktop/FishCam/logs/

### 6.9 Pre-deployment checklist

In order to minimize failures, it is recommended to go through the following steps before deploying the FishCam in the field.

• D-cell batteries at least 1.4 V.
• All wires properly connected and undamaged.
• Camera ribbon connector properly attached/not loose.
• Camera lens clean and free of debris.
• Buzzer’s 9 V battery connected.
• O-rings of the pressure housing clean and greased with silicon lubricant.
• Front-view window clean.
• Time on the FishCam clock is correct (check with the command “date” in an SSH terminal).
• RTC of the WittyPi is synchronized and ON/OFF schedule is operational.
• Buzzer rang after power turned ON.
• Files recording properly (check number of files recorded, file sizes and logs via SSH).

## 7 Validation and characterization

**Fig. 42:**
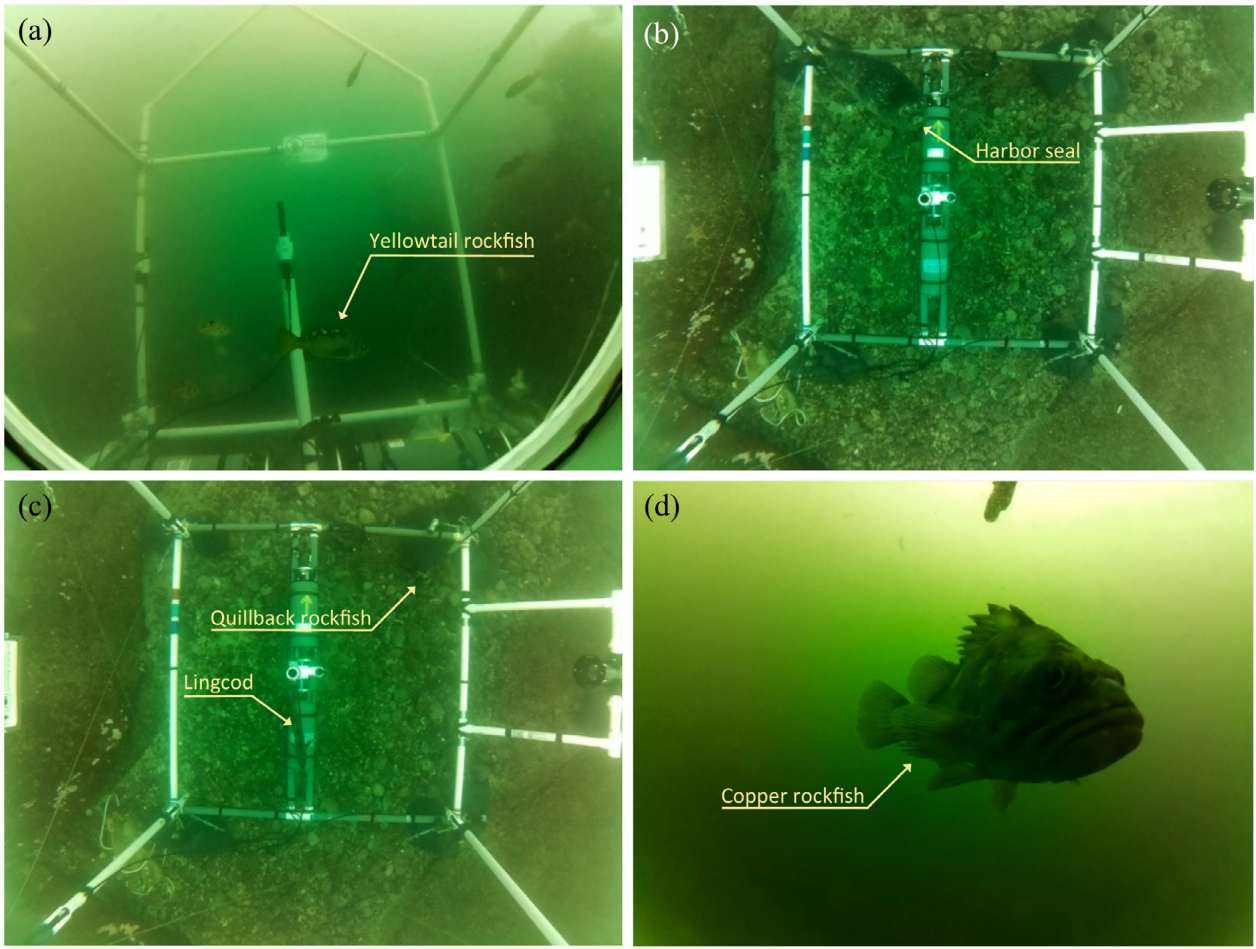
Examples of marine life captured in British Columbia waters by the FishCam: (a) yellowtail rockfish (*Sebastes flavidus*) at Ogden Point, Victoria, 10 m depth; (b) harbor seal (*Phoca vitulina*) at Hornby Island, 8 m depth; (c) quillback rockfish (*Sebastes maliger*) and lingcod (*Ophiodon elongatus*) at Hornby Island, 8 m depth; and (d) copper rockfish (*Sebastes caurinis*) at Mill Bay, 9 m depth. Note that the lower square of the PVC frame in panels (b) and (c) is of dimension 2 m × 2 m. The associated videos are in the suplementary multimedia component 2.

**Fig. 43:**
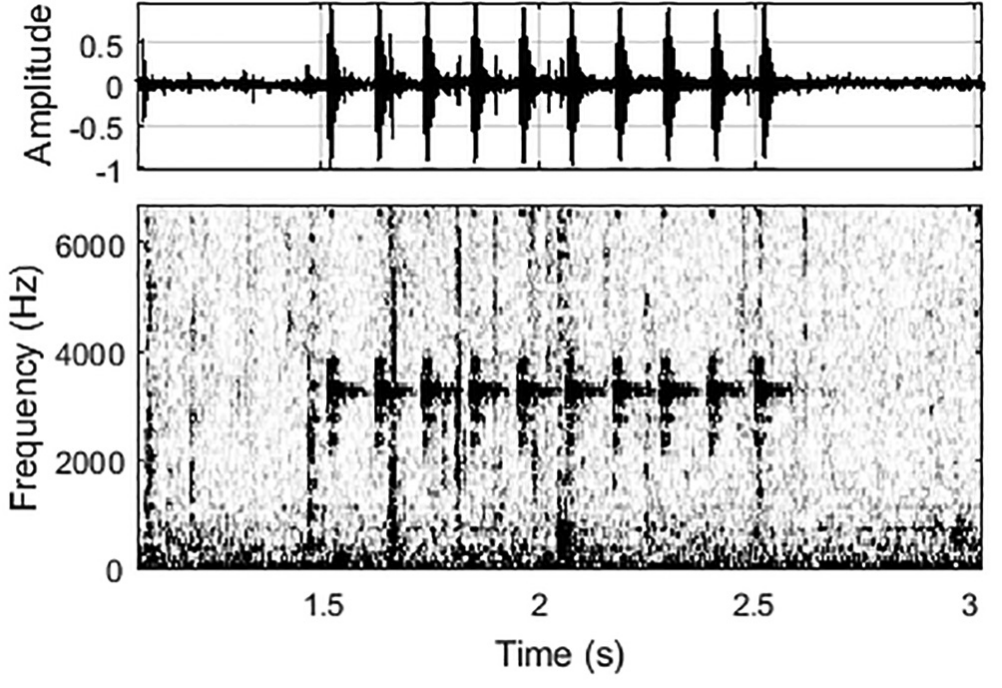
Waveform (top) and spectrogram (bottom) of a 3 kHz-tone sequence from the FishCam received by a hydrophone located 3 m away. The sampling frequency of the acoustic recorder was 32 kHz. The spectrogram was computed by Fast Fourier Transform using 512-sample Hanning windows overlapped at 95%. The corresponding video for this figure is in supplementary multimedia component 1.

Three FishCams were built and deployed at five sites on the east coast of Vancouver Island, Canada, from January to December, 2019, as part of the Fish Sound Project [14]. A total of ten deployments were conducted at water depths of 8–12 m (Table 1). One of the FishCams (FC-00) did not have enough ballast and was dragged by strong currents. It was found 6 months (180 days) later on a beach ~50 km away from its original deployment location. The electronic components were all intact and operational upon retrieval. However, the front-view window of the pressure housing was heavily scratched, which made this unit unusable for further deployments. The other two FishCams (FC-01 and FC-02) were deployed multiple times for durations of 8 to 14 days. In these deployments, the FishCams were attached either horizontally to the PVC frame shown in Fig. 3c near the seabed (e.g. Fig. 42a,d), or vertically to a larger PVC frame at 3 m above the seabed (e.g. Fig. 42b,c). For all deployments, the FishCams were configured to record video files during daylight hours only, at 10 frames per second and at a resolution of 1600 × 1200 pixels. The minimum and maximum duration of video recordings collected per deployment were 80 h (Armstrong Point) and 212 h (Mill Bay), respectively, with an average (± standard error) of 124 ± 11.6 h. This variability is largely due to variations in water visibility. Video files are compressed by the camera board during acquisition, which results in smaller file sizes in low-light environments and larger files in bright and clear conditions. The sixth deployment at Mill Bay occurred during a phytoplankton bloom (Fig. 42d), which resulted in poor light conditions and consequently smaller video files. In all cases (except the first deployment), the FishCams were still powered and running when retrieved, but their memory cards were full, indicating that even after 14 days, they were not battery limited, but memory limited. Using larger memory cards would increase the maximum deployment duration and/or the quality of video data collected. The resolution of the FishCam videos acquired during the ten deployments allowed us to successfully count and identify fish swimming in the field of view of the camera, and to observe inter- and intra-specific behaviors (Fig. 42). The sequence of 3 kHz tones emitted by the buzzer inside the FishCam pressure housings was loud enough to be detected by hydrophones located 3 m away, and provided successful synchronization of video and audio data (Fig. 43, Supplementary Multimedia Component 1).

**Table 1:** Deployments of the FishCam in the field.

Field trial #	Location	FishCam ID	Deployment date	Depth (m)	Minimum water temperature (°C)	Deployment duration (days)	Date of first recording	Date of last recording	Hours of video recorded
1	Armstrong Point	FC-00	2019-01-21	Variable	Unknown	~180	2019-01-21 06:28:46	2019-01-27 17:55:53	80.3
2	Ogden Point	FC-01	2019-05-03	10	11	8	2019-05-03 18:00:58	2019-05-10 13:35:19	163.2
3	Ogden Point	FC-01	2019-06-15	10	12	14	2019-06-15 15:22:00	2019-06-20 18:51:41	98.9
4	Ogden Point	FC-02	2019-06-15	10	12	14	2019-06-15 15:31:35	2019-06-21 18:21:33	117.3
5	Mill Bay	FC-01	2019-07-29	9	12	9	2019-07-29 15:40:24	2019-08-04 05:56:31	109.1
6	Mill Bay	FC-01	2019-08-18	9	15	14	2019-08-18 16:13:55	2019-09-02 01:36:16	212.2
7	Hornby Island	FC-01	2019-09-15	8	14	8	2019-09-15 16:01:23	2019-09-21 02:21:43	89.0
8	Hornby Island	FC-02	2019-09-15	8	14	8	2019-09-15 16:50:17	2019-09-23 18:16:42	127.0
9	Snake Island	FC-01	2019-11-28	12	11	13	2019-11-28 17:13:53	2019-12-05 14:21:44	111.2
10	Snake Island	FC-02	2019-11-28	12	11	13	2019-11-28 13:24:43	2019-12-06 23:11:50	132.4

The FishCam is an autonomous camera system that allows video and still images to be captured underwater over longer time periods than most camera systems currently available on the market. The design is simple, easy to build, and inexpensive (<500 USD). It has been used successfully in the field to non-intrusively observe fish in their natural habitat. Thanks to the flexibility of the Raspberry Pi board, the design is versatile and can easily be extended to fulfill specific needs such as, adding external lights or sensors (e.g. pressure, temperature). Both homemade and commercial pressure housings worked properly. The buzzer circuit was used successfully to synchronize the video data with acoustic data collected by nearby recorders. With the current design, the FishCam can record up to a maximum of 212 h of videos over a period of 14 days. The camera sensor used here is inexpensive and has a resolution appropriate for the water depths tested in the field (12 m).

The limitation of recording duration is due to the memory storage (200 GB) rather than the battery life of the FishCam. The storage size of microSD cards available on the market is increasing constantly and the cards are becoming less expensive. Therefore, it is expected that in the very near future, a larger microSD card could be used in the FishCam at no extra cost, overcoming current memory restrictions. Storing data on an external USB drive instead of on the microSD card has been tested in previous iterations of the FishCam, but was found to draw more power and consequently reduce the recording time. It was also found to be less reliable in the field since the USB connection can easily be damaged or disconnected due to the vibrations of the boat during transport, thereby compromising data collection. The supercapacitor used in the WittyPi can keep track of time without power for a maximum of 17 h, which restricts the maximum OFF time of the FishCam duty cycle. This can be overcome by connecting a 3 V button battery to the WittyPi board as indicated in the user manual from the manufacturer. During an early deployment, a small amount of water was found in the homemade PVC housing upon retrieval. This was addressed by reinforcing the seal of the front-view window with additional epoxy. Care should be taken when building the PVC pressure housing as small imperfections in this process can potentially lead to failure of the housing (the ready-made housing from Blue Robotics is more expensive but less prone to failure). Deploying the FishCam at deeper depths would require external lights or a more expensive camera sensor. Both pressure housing designs used for the FishCam worked as intended in the field. While the homemade PVC design from [15] is less expensive, the commercial housing from Blue Robotics is more reliable and versatile, allowing more external connectors and sensors. Due to the light weight of the FishCam and its PVC frame, it is important to add enough ballast during deployment to limit risks of losing the instrument with strong currents. All components of the FishCam were purchased in small quantities; larger orders of components could further reduce the cost of the FishCam. Such an approach is used successfully in other open-source projects such as the AudioMoth acoustic recorder [21]. Future developments of the FishCam could include the addition of an external piezzo transducer to emit more complex sounds directly in the water that could be used, for example, in animal behavioral response studies. One or more hydrophones could also be added to the FishCam using Raspberry Pi compatible sound acquisition boards [22]. The FishCam could find applications in a wide range of aquatic research. Many marine ecology research studies use baited remote underwater video (BRUV) to record fish diversity, abundance, and behaviour [1]. Many of these BRUVs typically have an autonomy of fewer than 10 h [23], [24]. A FishCam could be used in BRUV studies to expand the duration and spatial coverage of the monitoring effort. FishCams deployed in pairs could potentially be used as stereo-video systems for accurate fish size measurements [25], [26], although this may require the use of a different lens.

Advances in technology, along with the increasing popularity of open-source systems and software, allow researchers to build sophisticated research instruments at lower costs. These innovations can then be made accessible to a much broader demographic. Through this approach, new instruments are becoming available to the marine research community to monitor underwater environments over longer periods of time, over greater spatial scales, and at a minimal cost. Advanced electronic components, such as those used in the FishCam, become not only more accessible to the research community, but also to the general public. This encourages citizen science initiatives that have the potential to improve and expand ongoing research by scientists [27]. Our vision for the FishCam is to have applications in education, citizen science and ecological research. The FishCam could be built by students to teach them the basics of electronics, programming and environmental science, and be used in deployments by citizen scientists (e.g. recreational divers) and students, to acquire data to be analyzed by researchers to address ecologically-important questions. Such an approach offers a unique opportunity to engage with students and local communities to learn new skills that contribute directly to real-world research and conservation [28], [29], [30].

### 8 Summary

Capabilities of the FishCam:

• Maximum video capacity of 212 h over a period of 14 days.
• Duty cycles fully customizable.
• Acquisition of videos and/or pictures.
• Large field of view (110^°^).
• Wireless access and configuration.
• Possibility to add external sensors.
• Inexpensive and easy to build.

Limitations of the FishCam:

• Needs additional external lights for deployment in deep water.
• Basic electronics skills required (i.e., soldering).

## Human and animal rights

Not applicable.

## Declaration of Competing Interest

The authors declare that they have no known competing financial interests or personal relationships that could have appeared to influence the work reported in this paper.
